# Roles of Waste Glass and the Effect of Process Parameters on the Properties of Sustainable Cement and Geopolymer Concrete—A State-of-the-Art Review

**DOI:** 10.3390/polym13223935

**Published:** 2021-11-14

**Authors:** Ayesha Siddika, Ailar Hajimohammadi, Wahid Ferdous, Veena Sahajwalla

**Affiliations:** 1 School of Civil and Environmental Engineering, University of New South Wales (UNSW Sydney), Sydney, NSW 2052, Australia; 2 Centre for Future Materials, University of Southern Queensland, Toowoomba, QLD 4350, Australia; Wahid.Ferdous@usq.edu.au; 3 Centre for Sustainable Materials Research and Technology (SMaRT@UNSW), School of Materials Science and Engineering, University of New South Wales (UNSW Sydney), Sydney, NSW 2052, Australia; veena@unsw.edu.au

**Keywords:** waste glass, pozzolanic reactivity, cement, concrete, geopolymer, sustainability

## Abstract

Recent research has revealed the promising potential of using waste glass (WG) as a binder or inert filler in cement and geopolymer concrete to deliver economic and environmental benefits to the construction sector. However, the outcomes obtained by different research groups are scattered and difficult to compare directly because of isolated process parameters. In this study, the roles and impacts of WG and process parameters on the performance of WG-added cement and geopolymer concrete are critically reviewed. This study reveals that the chemical and mineralogical composition, and particle size of WG, mix proportion, activation, and curing condition of concrete are the most important parameters that affect the dissolution behavior of WG and chemical reactivity between WG and other elements in concrete; consequently, these show impacts on properties of concrete and optimum WG level for various applications. These parameters are required to be optimized based on the guidelines for high pozzolanicity and less alkali–silica reactivity of WG in concrete. This review provides a critical discussion and guidelines on these parameters and the chemistry of WG in cement and geopolymer concrete for best practice and highlights the current challenges with future research directions.

## 1. Introduction

Glass is a widely-used material due to its attractive appearance, transparency, ease of molding, high durability, and resistance to abrasion [1]. As a result of the high rates of glass production and use (around 130 million tons), over 100 million tons of waste glass (WG) are generated around the world each year, which makes up approximately 5% of the total solid waste generated per year in the world [2,3,4,5]. Only a part of containers and packaging WG is being recycled as raw material for new glass products, but the WG recycling process requires a large amount of energy, which may not be feasible due to its specific color and physical characteristics [6,7]. Other types of glasses, such as window panes, tempered, laminated, Pyrex, borosilicate glass, light bulbs, mirrors, glassware, and ovenware, are not used as raw materials for glass production and are considered contaminants [8,9]. For example, laminated glass has limited recyclability due to its unique and complex structure, which is formed by the strong adhesion of the polymer resin layer sandwiched between two glass layers [10,11]. WG from cathode ray tubes contains lead and is considered hazardous waste [12]. Therefore, the recycling process requires sorting and treating different WG separately [7,13], which makes the entire process complicated.

In 2019, the glass recycling rate in Australia was 57%, but the rest of the waste glass was discarded as waste [14]. In the UK, about 500,000 tons (28–31%) of WG are being sent to landfills yearly, and the remaining waste is recycled into white glass windows [15]. In Hong Kong, 90% of glass bottle waste is being dumped in landfills [16], and in Turkey, approximately 67% of WG (about 80,000 tons) is recycled [1]. The rate of glass recycling is highest in Europe (73%), and the recycling rates are lower in the US (34%) and Singapore (20%) [9]. In 2018, around 27 million tons of glass were recycled globally by glass industries, and the rest of the non-recyclable WG were landfilled [17]; therefore, the WG recycling sector needs special attention to prevent the loss of valuable material, avoid environmental degradation, and reduce the demand for landfills.

Concrete casting requires natural sand and natural stone aggregates, which are around 20–30% and 60–70% of concrete volume. Excessive extraction of natural sand and stone aggregates may also result in resource shortages [18,19]. Mining of natural river sand causes changes in the direction of river flows and alters hydrological strata and river bed levels [20]. In addition to cement production, concrete casting requires substantial energy, consumes natural limestone, and is responsible for 5–7% of the world’s total carbon dioxide emissions [21,22,23,24,25,26]. Therefore, research on the appropriate and sustainable substitutions of natural and conventional ingredients in concrete is essential. Glass contains amorphous silica and shows high pozzolanic reactivity, which is desirable in supplementary cementitious material (SCM) used in concretes, as it potentially enhances the chemical resistance and durability of concretes [9,27,28]. Depending on its type and fineness, WG can be used to replace different ingredients in concrete. WG can be used as SCM and aggregates in concrete, replacing ordinary Portland cement (OPC), natural fine and coarse aggregates [15,23].

Geopolymers are a class of inorganic aluminosilicate-binding materials with an amorphous or semicrystalline three-dimensional structure [22]. The production of a geopolymer is associated with the reaction of aluminosilicate raw materials with alkaline activators, and therefore requires a substantial source of silica [24]. WG powder can be used as a precursor, fine aggregate, and activator in the production of cement-free geopolymer binders and geopolymer concretes [16,29,30]. Current applications of WG concrete (WGC) include precast concrete elements for building construction, paving blocks, marine structures, foamed concrete, geopolymer concrete, and geopolymer foams for lightweight structures [15,22,31]. The physical properties, chemical composition, amount, and particle size of WG used in concrete are the main parameters that control the performance of WGC [6,10]. Lightweight but high-performance concrete can be developed using WG as SCM, precursors, or aggregates [1,32,33,34]. For example, Adaway and Wang [7] observed a 6% higher compressive strength for concrete with 30% glass aggregates compared to the control specimen. As reported in previous research [35], around 30% improvement in strength and high interfacial bonding strength can be developed by using WG aggregates in geopolymer concretes. Alkaline characteristics and high silica dissolution from fine WG powder are the main parameters for strength development in geopolymer concretes.

Replacement of cement by WG in concrete is beneficial in terms of the reduction of CO_2_ emissions and construction costs. As reported by Islam et al. [36], it is possible to reduce cost by approximately 14% and CO_2_ emissions by 18% by replacing 20% cement with WG powder in a concrete structure. Moreover, the CO_2_ emissions related to the production of sodium silicate solution can be minimized through the incorporation of WG in geopolymer concretes. However, the most critical consideration is the antagonistic characteristic of WG in concrete—which, on the one hand, improves mechanical properties due to the pozzolanic effect, and on the other hand, exhibits detrimental alkali–silica reaction (ASR), which reduces the durability of concrete [10]. Therefore, there is a continuous quest for mitigation of the negative ASR impact and improvement in the performance of WGC.

This study aims to provide a fundamental background on the use of WG in cement and geopolymer concretes and to describe its comparative performance with conventional materials. There are some review studies on WGC [37,38,39], but most have been focused on the properties of cement-based WGC with any specific replacement type. These previous studies focused directly on the properties of WGC, where the correlations between the process parameters and role, reactivity of WG, and its constituents within concrete are still unclear. Additionally, the processing parameters are scattered in different research, which needs to be optimized for best practice. This review presents the fundamentals and science behind using WG as a binder, precursor, and aggregate in concrete. It explains the chemical composition and physical properties of WG, along with their effects on the physical, microstructural, and mechanical properties of concrete, according to existing practices and guidelines. Additionally, the guidelines about the crucial processing parameters are correlated with the reactivities of WG and the performance of concrete. The concurrent documentation of the uses of WG in cement and geopolymer concrete will be helpful for readers and practitioners. Herein, we summarize the current knowledge in this field and describe future opportunities for research to promote the use of WG in the construction sector.

## 2. Glass Powder as a Supplementary Cementitious Material

### 2.1. Properties of Typical Glass Powder

**Figure 1:**
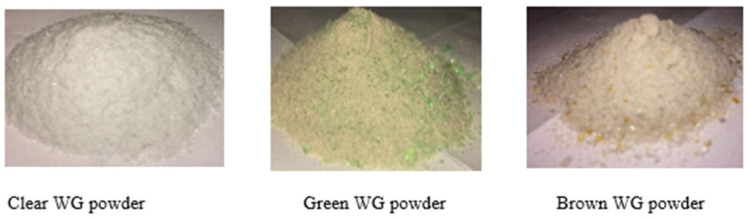
WG powder derived from waste glasses [43].

Glass is inorganic, non-metallic, hydrophobic, incombustible, and brittle, although it is highly ductile at high temperatures [40]. It can be classified into 24 types, but the major types are soda-lime, fused silica, lead, vitreous silica, borosilicate, alkali silicate, aluminosilicate, germanium oxide, and barium glasses [28,29,30]. It can also be flat or shaped into bottles, cathode ray tubes, or lamp glasses, depending on its production and use [15]. Typical WG powder is shown in Figure 1. The WG, when processed into fine particles, fulfills the requirements for the physical, mechanical, and chemical characteristics of Class F and Class C pozzolanic materials according to the guidelines provided by ASTM C 618 standards [27]. Therefore, it can be incorporated into concrete as SCM. Additionally, the glass particles’ sizes are decided based on the roles of WG in concrete. In general, as SCM, particle sizes should be below 600 µm. For fine and coarse aggregate replacement, WG particles’ sizes are selected below 4.75 mm and 14 mm, respectively. The density and surface area of WG particles can be changed by treatments such as washing, milling into the required size, and gradation. For the detailed milling and grinding process, the readers are redirected to [40,41,42].

**Figure 2:**
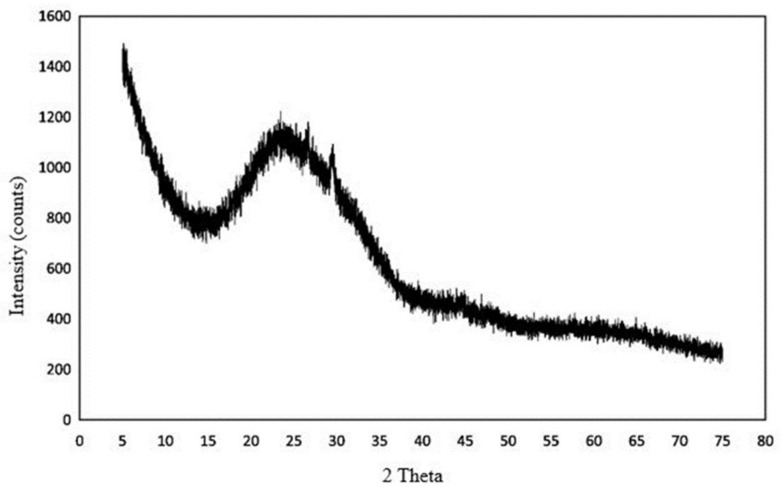
X-ray diffraction pattern for glass powder (windshield glass) (reformatted from [10]).

Typically, the density of glass is approximately 1600–1700 kg/m^3^, with a specific gravity of 2–2.6. However, the properties of different glasses vary according to their compositions. The primary constituents of glass are silica, lime, and soda [8,15]. Thus, glass possesses a distinct melting point. Other ingredients, such as flux salts, inorganic colorants, hardness, and durability improvement agents, are added following various production methods and applications [8]. Na, Al, Ca, Fe, S, and K oxides are present in glass. Typical chemical compositions of WG obtained from different sources are listed in Table 1. The variation in chemical composition in glass is minimal with color variation but significantly dependent on its production process and application [44]. Each chemical constituent in glass plays a distinct role in concrete, which affects the properties of the final composite. Typically, the glass particle is purely amorphous (Figure 2) [38,45]. The high amount of amorphous SiO_2_ makes glass suitable for use as a pozzolanic material in concrete. The other components, such as CaO and Al_2_O_3_, enhance the reactivity and hydration of glass powder; consequently, a high amount of C–S–H and Al–O–Si bonds are developed depending on the activation and curing conditions [23,44,46]. On the contrary, the presence of P and Zn in WG reduces the hydration rate and can act as hydration retarders in cement-based concrete [16]. Furthermore, glass is highly alkaline, with a pH of approximately 11 [27]. Additional alkali oxides such as Na_2_O enhance the alkalinity, which changes hydration products such as Na–Al–S–H [46,47,48]. Details of the impact of ingredients that have been considered in the current literature are discussed in the following sections.

### 2.2. Reactivity of Glass Powder

**Figure 3:**
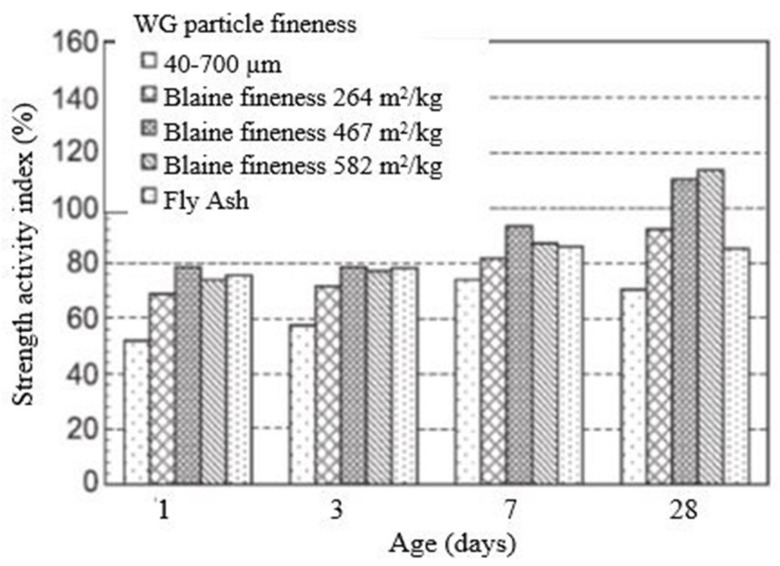
Strength activity index of WG powder with varying fineness [52].

The main reason for using SCM in concrete is its pozzolanic reactivity, which is very important because, through this reaction, a high amount of final hydration calcium-silicate hydrate (C–S–H) products are formed in concrete. Pozzolanic reactivity is directly related to the amount of reacted CaO, Ca(OH)_2_, in the sample and the production of C–S–H products [50]. Being amorphous and containing a high amount of SiO_2_ (Table 1) and some CaO, WG shows high pozzolanic reactivity [16]. In concrete, pozzolanic reactivity depends on the particle size and amount of WG and the curing conditions. Pozzolanic reactivity of WG powders is around 70–110% [51,52,53] at 28 days, which meets the criteria for pozzolanic materials by ASTM C 618 [54]. Typical pozzolanic behavior of WG is presented in Figure 3 [52]. This figure shows how the pozzolanic index of WG varies with its fineness and age for a cement replacement level of 20%. As shown in Figure 3, the pozzolanic reactivity of the glass powder increased with its specific surface area. With increased fineness and amorphous content, the pozzolanicity of glass powder can be even higher than the pozzolanicity of commonly used fly ash. The finer WG powder has a high silica release rate; it has a higher chance of reaction with Ca^2^+ and OH^−^ ions in an alkaline medium and forms a higher amount of C–S–H. The recommended particle size of the WG powder is 75 µm for high pozzolanic reactivity, and the finer powder is more pozzolanic [44,55]. Moreover, these size group WG particles show no unfavorable ASR expansion in concrete [56]. The maximum WG particle size is ≤600 µm to avoid the ASR expansion either by WG SCM or aggregates in concrete [51]. Details of the ASR parameters for WGC are discussed in Section 4.1.

**Figure 4:**
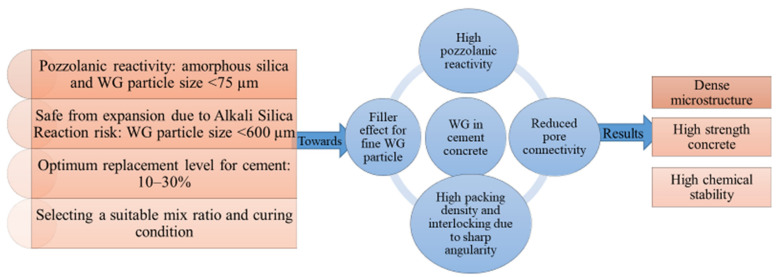
Selection guidelines and the optimum mixing parameters of WG to develop high-performance concrete [4].

WG, when being used below 10% of cement, releases a small amount of silica during the early age of hydration, which shows very little pozzolanic reaction [57,58]. Hendi et al. [53] used WG particles below 1 µm with a Blaine fineness of 800 m^2^/kg, and the cement replacement level was 20%. The authors reported that the pozzolanic reactivity of the WG powder was 61% and 70% at 7 and 28 days, respectively. Beyond a 30% cement replacement level, a lack of CaO can result, which also hampers the pozzolanic reactions. To ensure early-age pozzolanic reactivity and the production of sufficient C–S–H gels, the Ca/Si ratio should be around 0.81–1 [59]. Therefore, based on the particle size, the amount of WG influences pozzolanic reactivity in concrete (Figure 4). Furthermore, raised curing temperature (around 50 °C) and heat treatment improve silica dissolution and ion transformation during hydration of WG [60] and contribute to the high strength of concrete. For heat-treated WG powder (specific surface area 800 m^2^/kg), Hendi et al. (2019) [53] found a 126% pozzolanic index. With a longer curing period, more C–H products and silica would be available for the reaction, thus increasing the reactivity of WG.

Overall, the pozzolanicity of WG depends on its amorphous nature, silica dissolution rate, available Ca(OH)_2_ in the solution, alkaline environment, curing time, and temperature. However, the only factors that have been extensively studied in the literature are the impact of WG particle size and the percentage of WG in concrete. The effect of other chemical constituents in glass has not been completely investigated and discussed in the literature. There are some contradictions about the impact of color of WG on the properties of concrete. For example, amber and clear glasses are reported to show higher pozzolanic reactivity than other colors [43,50], although the reason for this difference has not been clarified. This could be attributed to the high amorphous content in those glasses.

By washing in an alkaline solution or applying heat treatment, the pozzolanic reactivity of WG can be increased because both methods reduce impurities and increase the rate of silica dissolution [48]. However, it is not clear which chemical and physical variations have a key influence on the pozzolanic reactivity of WG and the optimum treatment conditions. However, to ensure the optimum rate of silica dissolution throughout the entire strength development period, well-graded WG particles are essential. Glass particles could have microcracks and entrapped voids within them due to unusual processing, which is one of the reasons for ASR expansion [61]. Currently, no research has investigated customized WG processing and particle size distribution to optimize their reactivity in concrete. Further investigation of the impact of the size distribution and grinding of glass is required to understand the optimum processing and gradation of WG for high-performance concrete.

### 2.3. Hydration Characteristics of the Glass Powder

**Figure 5:**
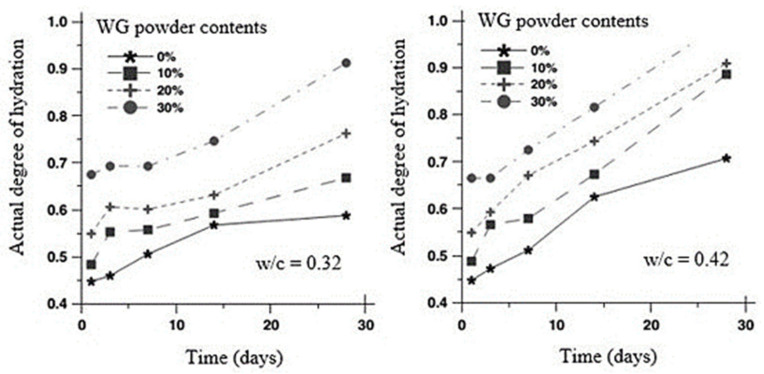
Degree of hydration in cement pastes with various amounts of WG powder [62].

**Figure 6:**
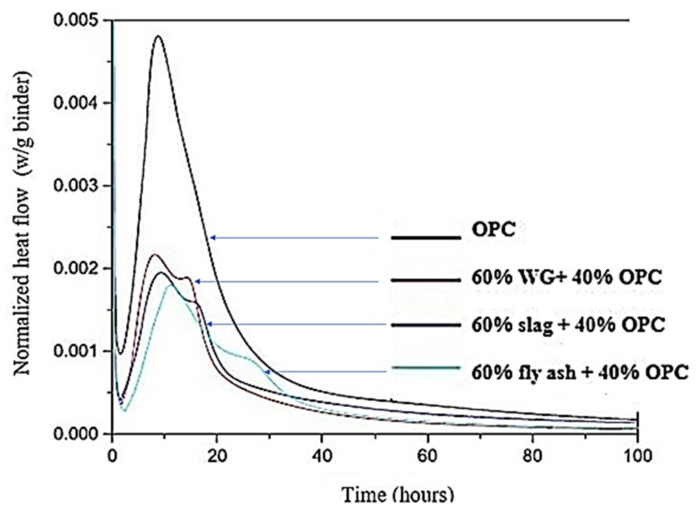
Heat flow during hydration of different binders [63].

Hydration of the binder is an important stage for the development of the microstructure and strength of concretes. The degree of hydration of cement paste generally increases with WG powder content (Figure 5) [62]. The time of the first heat flow peak observation in OPC + 60% WG powder is shorter than that in OPC + 60% slag or OPC + 60% fly ash, as shown in Figure 6 [63]. This is due to the higher silica dissolution in WG powder paste compared to slag and fly ash, which creates a higher chance of reaction with Ca(OH)_2_. However, compared with pure cement, the induction time was very close. There was also a slight increase in the induction period with an increasing amount of WG in the binder [64]. The alkaline medium and the amorphous SiO_2_ content in the WG powder induce pozzolanic reactivity [52]. Thus, a high number of reaction products are developed that cause substantial heat release during hydration in concrete, and the heat flow is generally much higher than that of other traditional SCM, such as fly ash and slag (Figure 6). This occurs in the early stage of hydration due to the less reactivity of silica in fly ash and slag compared to in glass [63]. The production of a high amount of portlandite (C–H) and C–S–H is an indication of enhanced hydration and improved strength development in cementitious pastes with WG powder [42,50].

However, a retarding effect at early-stage hydration was observed in cement pastes with WG powder that had P and Zn elements [16]. These components in WG can combine with free SO_4_^2−^ and OH^−^ ions in the solution and produce ZnSO_4_.nH_2_O, CaSO_4_.nH_2_O, and other SO_4_^2−^ products, which can delay hydration reactions [16]. The retardation rate in hydration can be reduced by using WG powder with particle sizes below the threshold limit of the pozzolanic requirement. As reported in the literature, glass particles below 75 µm show a higher hydration degree [44,55,65]. Another matter of concern is the flash and false setting of the binder due to its poor hydration. The presence of Zn and a high alkali content (Na, K) in the WG powder can trigger the flash setting of the binder [66]. A proper w/c ratio (0.45–0.50) can positively influence the degree of hydration and setting. However, the dilution of constituents may also delay the reaction when the w/c ratio increases beyond the optimum level (0.45–0.50). Previous reports claim that glass possesses water-repellent characteristics, which may delay the setting of composites when they are agglomerated [67]. Due to the limited data and conflicting information in the current literature regarding the impact of WG on setting time, understanding and guidelines are still insufficient. Further investigation is required to clarify the influence of glass ingredients on hydration and the setting time of cementitious pastes. A thorough investigation is required to properly correlate the degree of hydration and pozzolanic reactivity of WG powder in concrete because there is currently no existing research in this field.

### 2.4. Properties of Concrete with Waste Glass as a Supplementary Cementitious Material

#### 2.4.1. Physical Properties of Fresh and Hardened Concrete

##### Workability of Concrete with WG as SCM

**Figure 7:**
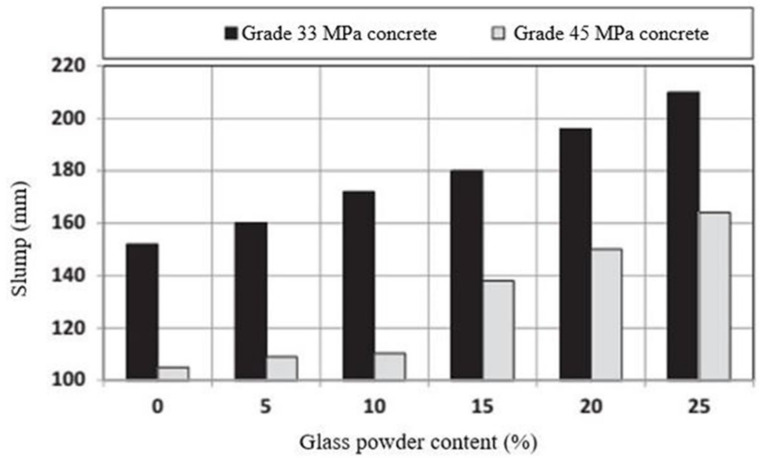
Slump values of concrete with WG powder (particle size ≤ 15 µm) [27].

Slump value is a measure of the workability of concrete and is controlled by the roughness, fineness, and amount of WG, regardless of the mix proportion, mixing technique, and water/cement ratio in concrete. Based on the literature, a WG particle of size 45–150 μm has a positive effect on the slump value of WGC. Because of the smooth surface and low water absorption of fine WG powder (≤150), concrete shows a higher slump value with increasing WG powder content (Figure 7) [27]. With an increasing amount of WG, the workability of concrete increases regardless of the grade of concrete. For cement replacement by WG (<75 µm) of 0–30%, the slump of the concrete mix can be increased by 130–190 mm [68]. Due to the lower specific surface area of WG powder compared to OPC, less water flow is achieved, which improves the slump value. Moreover, in fine WG particles, the angularity of the WG does not lead to friction against the flowability of the concrete, and these are suitable for producing self-compacting concrete.

WG of particle size around 45 µm does not improve the workability compared to ordinary OPC concrete [69], as the same amount of water is required by cement and WG powder of this size. For WG particle sizes less than 10 µm, a significant decrease in workability was observed for a 20% replacement of cement in concrete [41]. Workability decreases due to the higher water demand by WG because of the higher specific surface area of the WG particles compared to OPC.

However, due to the high cement dilution and the formation of a low amount of hydration products in the early stages of WG hydration, high flowability is observed in concrete [27,40]. On the contrary, for flash and false settings, a high number of hardened products may be developed during mixing and casting, which is undesirable for both the workability and strength of concrete [50,70]. The poor geometrical shape, high aspect ratio, and angularity of WG enhance the reduction in concrete’s workability, resulting in a poorly consistent concrete mix when WG is used beyond the optimum level of 30% [1,7,71]. Segregation and bleeding may also be severe in WGC due to the flat and flaky edges of WG and can be aggravated with an increasing amount of WG and its particle size [41,71,72,73]. In summary, the main factors that affect the workability of WGC are particle size and the amount of WG in concrete, regardless of the other factors related to the concrete mix.

##### Density of Concrete with WG as SCM

**Figure 8:**
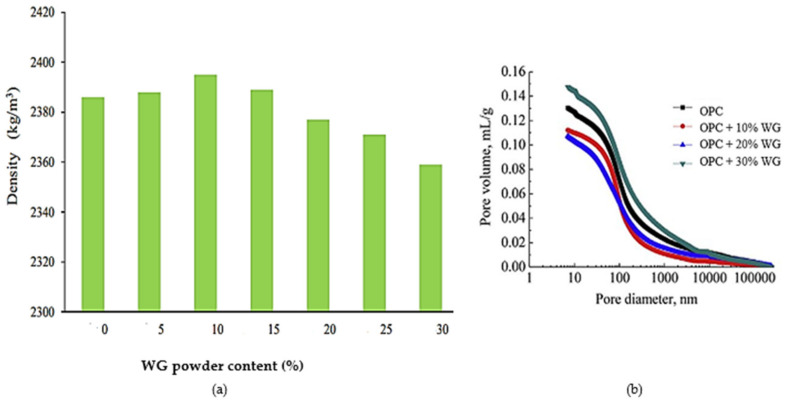
Density and porosity of WG powder-based concretes. (**a**) The density of concrete with WG powder (particle size < 100 µm) [75]. (**b**) The porosity of concrete with WG powder (Particle size 8–110 µm) [58].

Pozzolanic reactivity and filler effects are the two most important roles of the WG particle packing density and porosity reduction of cement concrete. The density of concrete varies according to the fineness, amount, and type of WG. Figure 8a shows a significant increase in concrete density with up to 10% cement replacement by WG powder. He et al. [58] found improvement in the density of concrete using 20% WG powder (0.8–110 µm) (Figure 8b). At a low replacement level (<20%), WG powder performed a filler role that reduces the voids between particles within concrete. A significant amount of hydration products (Ca(OH)_2_ and C–S–H) are formed, and the microstructure of concrete is densified with the high pozzolanicity of WG powder when added at the optimum level of replacement (20–30%) [16]. The density of concrete also increases due to the higher unit weight of fine WG powder (<75 µm) compared to that of cement [74].

Beyond the optimum level (30%), the addition of WG causes a reduction in density and an increment in the porosity of concrete. This is because the production of CH and C–S–H products reduces due to the fewer opportunities for pozzolanic reactivity of WG and reduced amounts of CaO [57,58]. Moreover, the unreacted WG causes agglomeration and creates voids within the concrete that reduces density. Du and Tan [57] showed that the control concrete (without WG powder) contained 14.3% porosity, whereas the concrete with 60% WG powder (0.5–100 µm) had 16.6% porosity. However, compared to the WG addition level, the porosity was minimal. Therefore, highly compact concrete can be developed with minimal porosity using WG as an SCM.

##### Microstructure of Concrete with WG as SCM

**Figure 9:**
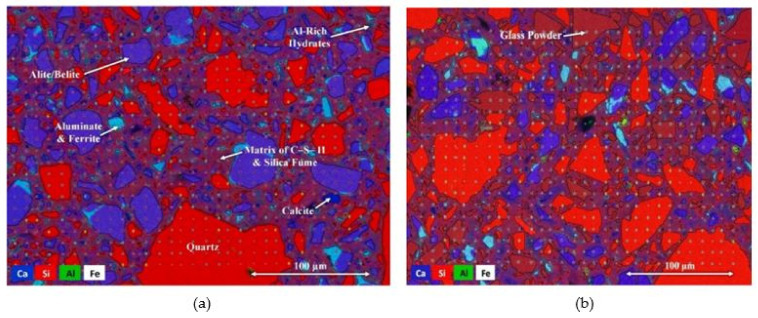
Chemical mapping of the surface in an ultra-high performance concrete (specimen showing the different anhydrous and hydrous phases) (**a**) without WG; (**b**) with 30% WG (12 µm particles) replacing cement [76].

Scanning electron microscopy (SEM) images of a specimen are generally used to show the internal pores, connectivity of pores, and uniformity of final reaction products in concrete. The microstructure of WGC depends on the particle size, mix ratio, and pozzolanic reactivity of WG. Finer and highly amorphous silica containing WG is suitable for producing a denser concrete micro-structure. Glass particles (<1.18 mm) act as microfilters at the early age of curing, and at low temperatures, their reactivity is low. WG particles of size below <75 µm show high filler and pozzolanic activity within the concrete and help to increase the density of concrete with a dense microstructure of the hardened matrix [44,55]. As found in ultra-high-performance concrete (Figure 9a,b), when 30% cement is replaced by WG (12 µm particles), the WG particle creates a denser skeleton, where the spacing between anhydrous inclusions and the thickness of the zone of hydrates are reduced [76]. The reactive surface of WG enhances the bond with the surrounding mortar and thus gives improved macro-scale strength compared to a matrix with similarly sized inert particles [76].

**Figure 10:**
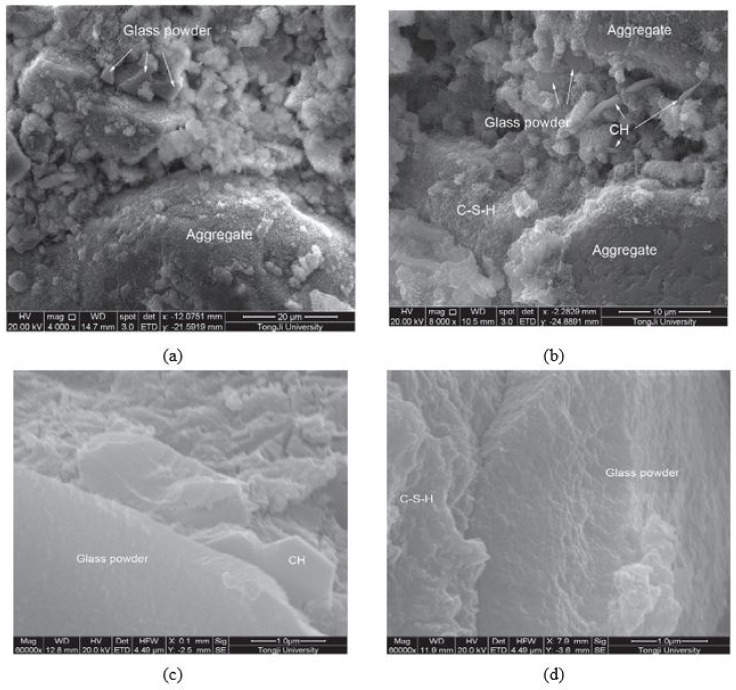
SEM images of the cementitious paste containing WG powder at different ages (**a**) 80 °C at 1 day; (**b**) Microwave curing at 1 day; (**c**) Normal curing at 28 days; (**d**) 80 °C steam curing at 28 days [77].

With the required fineness of WG and proper curing conditions, a dense microstructure of concrete can be formed with an optimum level of WG. This is evident in the research of Kong et al. [77]. As depicted in Figure 10a–d, the inclusion of 30% WG powder only showed a filling effect to produce a dense cementitious matrix in one day of age. The increased curing temperature is effective in increasing the pozzolanicity of WG powder, which is useful for producing CH and C–S–H products on the first day of age (Figure 10b). The porosity of concrete is reduced due to the formation of these products and the filling effects of WG powder, thus resulting in a dense microstructure of the hardened matrix.

Further, by room temperature curing for up to 28 days only, a low amount of hydration products was captured, but for steam curing at 80 °C temperature, a remarkably dense matrix is produced with significant C–S–H products [77]. These findings and the depictions (Figure 10a–d) clearly show the high pozzolanic reactivity and filler effects of WG powder at maintained conditions. However, the microstructure of the concrete matrix could be hampered by high temperatures, and voids could be generated due to the degradation of transitional hydration products.

#### 2.4.2. Mechanical Properties of Concrete with WG as SCM

The chemical composition and fineness of WG control its pozzolanic reactivity and filler effect, which are the main factors in producing high-strength WGC [15,27]. The strength of concrete is dependent on the chemical composition of WG powder, which is different for different colored glass because of the distinct type and production of glass [78]. Every kind of WG contains silica of around 60–80% [71]. The most important factors are the amorphous nature of the SiO_2_ and other ingredients (CaO, Al_2_O_3_, Na_2_O) that affect the pozzolanic properties of the WG. For example, neon glass contains around 22.6% CaO and 68.2% SiO_2_; thus, it yields 9.5% greater compressive strength in WGC than green glass, which yields 13% cement replacement [43].

**Figure 11:**
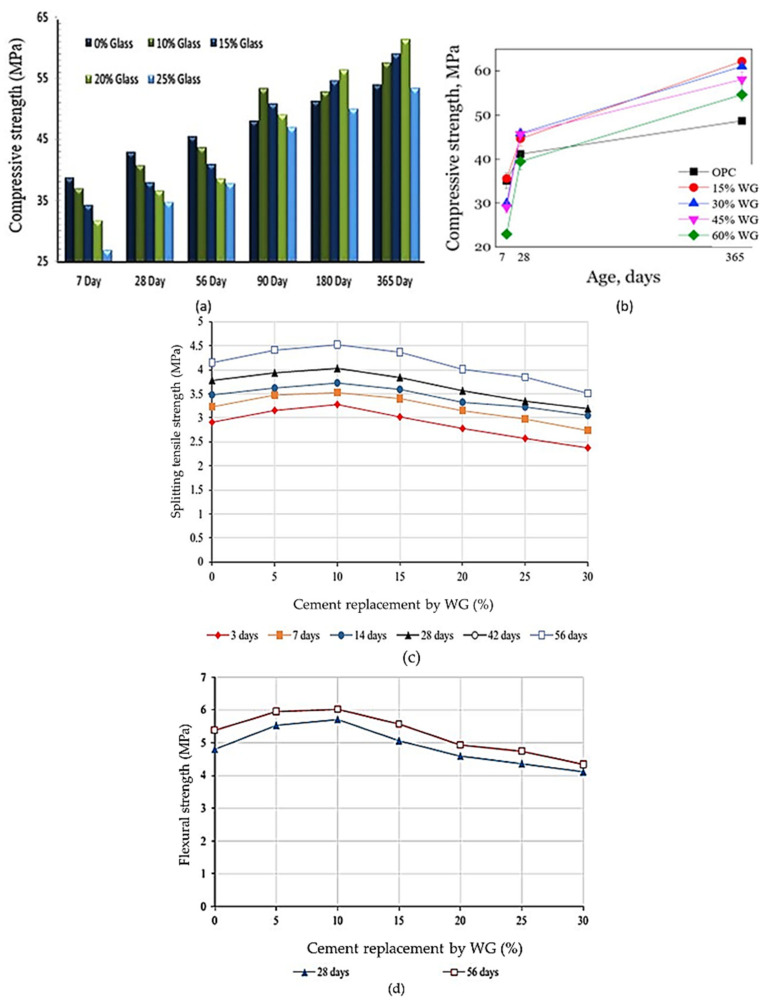
The variation in the strength of concrete with WG as SCM. (**a**) Compressive strength of concrete with WG powder (Particles < 75 µm) in replacement of cement [36]. (**b**) Compressive strength of WGC with WG powder (particles < 120 µm) as SCM [57]. (**c**) Splitting tensile strength of concrete with varying content of WG (Particles < 100 µm for cement replacement) and curing periods [75]. (**d**) Flexural strength of concrete with WG (Particles < 100 µm for cement replacement) [75].

The strength of concrete increases with the increasing fineness of WG [57,58,79]. The fine WG powder (particles 600–150 µm) acts as a filler, redistributes, and refines pores within the concrete matrix [66]. Finer WG powder (particles < 150 µm) shows better pozzolanic reactivity and filler effect, thus helping to produce more C–S–H products and a dense microstructure while developing more strength [79]. Kim et al. [80] observed approximately 22% and 11% improvement in mortar compressive strength after replacing 10% cement with WG powder of 5 µm and 12 µm particle size, respectively. Omran and Tagnit-Hamou [81] reported a 35% improvement in the splitting tensile strength of concrete at the 28-day curing age when WG powder (particle size < 40 µm) was used to replace 20% cement. The strength development rate in WGC is higher during an extended curing period [36,82] (Figure 11a–d). At a very early age (0–14 days), WG only shows micro filler activity and forms a minimal amount of hydration products. For a curing period between 28 and 90 days, the rate of strength development in the WGC specimens was found to be higher than that of the control specimens. Pozzolanic reactivity and strength development in WGC can occur up to 2 years, where no strength loss occurred up to 7 years [41,58,82]. With increased curing temperature, the pozzolanic reactivity of WG increases, and strength improvement occurs in WGC. For instance, mortars with 25% WG replacing OPC cured at 50 °C gained around 25% higher compressive strengths than those cured at 23 °C for 91 days [60].

**Figure 12:**
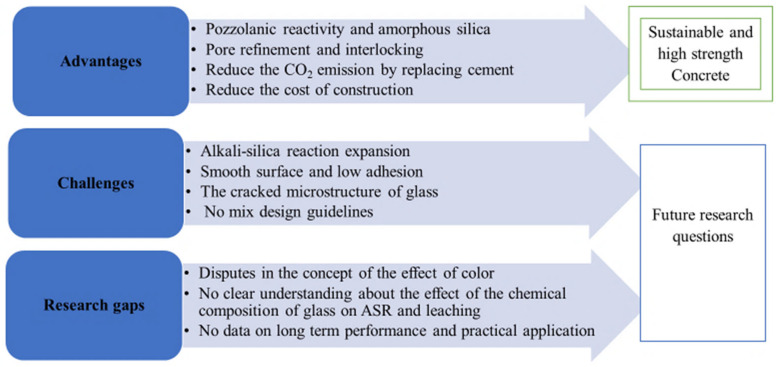
Advantages, challenges, and knowledge gaps of using WG as SCM.

However, agglomeration of unreacted silica can delay the pozzolanic reaction, and accelerated ASR may occur within the concrete when more than 30% of cement is replaced by WG [27,57,58]. Further, a weak interfacial transition zone is formed due to the low adhesion of glass and cement paste [83,84]. Typical relationships of WG amount and compressive strength, splitting tensile strength, and flexural strength of WGC are shown in Figure 11a–d. Some investigations of the mechanical properties of WGC are listed in Table 2, and the overall findings and research potential are shown in Figure 12. Table 2 demonstrates that the addition of WG as a construction material is beneficial for the concrete industry. There is a linear relationship between compressive strength and the splitting tensile strength of the WGC. The variation in compressive strength and splitting tensile strength of concrete with the variation in WG particle size and replacement level follows a nearly similar trend, regardless of the other compositions and parameters. Considering all the mechanical strength properties of WGC, the optimum level of WG as SCM is between 20–30% [51,75]. Additionally, the processing of WG powder has a significant impact on enhancing the bond strength of WG and cement paste [48], but this issue has not been considered in previous studies. To enhance the bond strength in WGC and interlocking between WG and cement paste, alkali solution, low-cost polymer, natural, or recycled waste fibers can be applicable. This issue needs to be investigated for future applications.

## 3. Glass as an Aggregate in Cement Concrete

### 3.1. The Role of Glass as an Aggregate in Concrete

Due to its high silica content, if properly graded, WG powder can fulfill the requirement for fine aggregates in concrete [31]. Therefore, ground WG is suitable for use as a replacement for natural sand aggregates in concrete. Due to the similar chemical compositions of natural river sand (Table 3) and WG (Table 1), similar behavior was observed when used in concrete. However, as a fine aggregate replacement in concrete, WG offers features such as filler activity, pozzolanic reactivity, and expansion due to alkali–silica reaction. Fine WG (<2.36 mm) shows filler activity in concrete and refines pore size [55,72]. The filler activity, pore refinement, and pozzolanic reactivity of WG aggregates increase the fineness of the WG (<600 µm). The CaO content in most WG is comparatively less than that in river sand; however, due to the higher pozzolanic reactivity of WG, it releases more silica and helps produce more C–S–H gels [87,88]. Therefore, WG aggregates accelerate hydration reactivity and create a dense and high-strength interfacial transition zone (ITZ).

WG glass with particle sizes larger than 4.75 mm is considered coarse aggregates in concrete and is typically sized up to 16 mm [89]. These types of WG aggregates are being used to replace the stone/brick chips in concrete to impart large volumes and mechanical strength [89]. Coarse WG particles do not show any pozzolanic reactivity, but due to their angularity and edges, WG particles help interlock the aggregate and cement paste with high bond strength. However, very limited studies have considered the use of WG coarse aggregate in concrete because of its unusual properties.

### 3.2. Properties of Concrete with Waste Glass Aggregates

#### 3.2.1. Physical Properties of Fresh and Hardened Concrete

##### Workability of Concrete with WG Aggregates

WG aggregates are generally angular, with a rough texture and sharp edges. When used to replace coarse and fine aggregates, these rough particles prevent the flow of concrete because of the high friction between them [7]. Typically, WG particles of 200 µm and greater size have a negative effect on concrete’s slump [90]. The friction and sharp edges of WG aggregate increase with increasing particle size; thus, the slump value of concrete reduces. On the contrary, the slump of concrete increases due to the low water absorption of WG compared to ordinary stone chips and river sand [91]. Further, the smooth surface of the ground WG can increase the flowability of concrete. For this reason, a 4% improved flow value of concrete was reported by Topcu and Canbaz [1] when 60% coarse aggregate was replaced by a 4–16 mm WG cullet.

##### Density and Microstructure of Concrete with WG Aggregates

**Figure 13:**
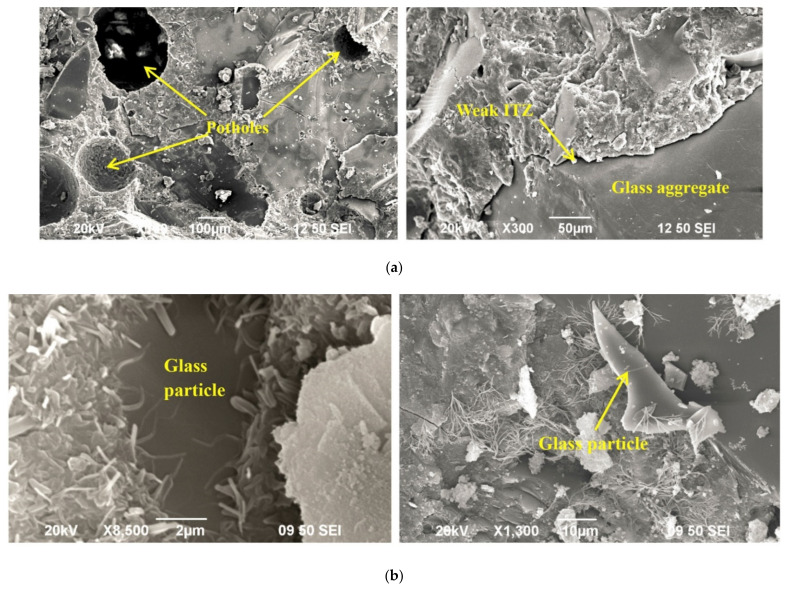
Microstructure and ITZ of mortar with WG aggregates [90]. (**a**) WG particle mean diameter of 204 µm. (**b**) WG particle mean diameter 28.3 µm.

The size of the WG in concrete is an important parameter that substantially controls the properties of concrete. The fine WG powder helps refine the pore size and divide the ITZ into a very thin layer, which increases the density of the concrete [91]. However, density of concrete decreases with the increasing particle size (>150 µm) of WG. A weak and porous ITZ is formed due to the lower pozzolanicity of coarse WG particles, and transitional C-H links are visible (Figure 13a,b) [90,91]. With a 20% WG aggregate (mean particle size around 204 µm), a weak and porous ITZ is visible in cement mortar, and at up to 90 days of curing, a significant number of unreacted particles are observed [90]. On the other hand, a 28.3 µm WG particle can produce fibrous hydration products, which makes the composite denser and stronger (Figure 13b).

Further, due to the lower specific gravity of WG compared with that of conventional stone aggregates, the density of concrete reduces when WG (Particle size > 2.36 mm) is used as coarse aggregates [89]. For a 25% replacement of sand by coarse size WG (particle size 150 µm–5 mm), the density of concrete was reduced by 25–30 g/cm^3^ [92]. Moreover, the rough geometrical shape of coarse WG can entrap voids within the paste, decrease the unit weight, and increase the porosity of the concrete [1]. Fewer hydration products may also result due to the higher ASR susceptibility of WG with increasing particle size, consequently forming a porous microstructure. On the contrary, the high angularity in WG aggregates may result in high interlocking with adjacent cement paste, resulting in a compacted and strong ITZ with up to 75% replacement of coarse aggregates [93]. Therefore, the angular shape of WG particles is also an important parameter for replacing coarse aggregates.

#### 3.2.2. Mechanical Properties of Concrete with WG Aggregates

**Figure 14:**
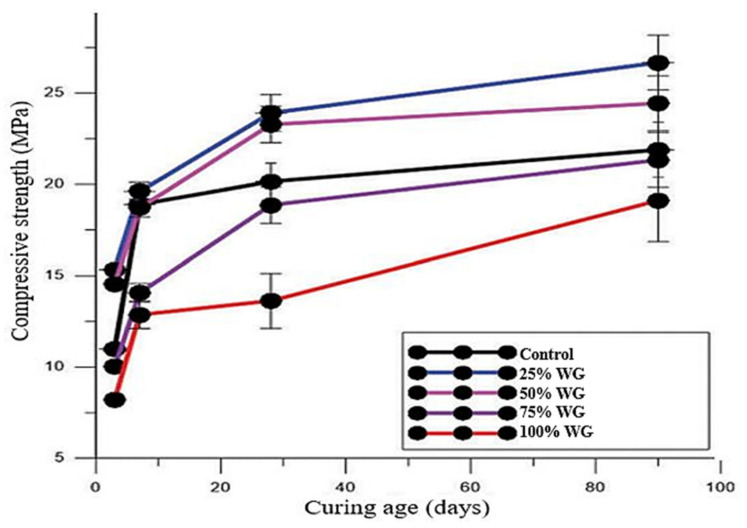
Compressive strength of concrete with WG aggregates (particle size 0.8–5 mm) in the replacement of sand [84].

Fine WG aggregates refine and redistribute pore sizes and strengthen the ITZ of concrete; thus, within finely ground WG, the strength of WGC increases. Olofinnade et al. [84] replaced 100% sand with WG of particle size 0.8–5 mm and found that, with up to a 50% replacement level, the strength of concrete was significantly improved compared to the control concrete (Figure 14). Ismail et al. [51] reported that the flexural strength of concrete at a 28-day curing age was improved by 3.57%, 6.96%, and 11.20% with the addition of 10%, 15%, and 20% WG (particle size 0.6–2.36 mm), respectively. The fine WG aggregates (<600 µm) participate in the hydration of binder and produce more reaction products, which improves the concrete strength. Due to the filler and pore refinement characteristics of fine WG, a high interlocked bond is developed, resulting in uniform stress distribution. However, with increasing particle size, the pozzolanicity of WG decreases, a lower amount of SiO_2_ dissolution occurs, and consequently, less C–S–H production takes place. Moreover, due to ASR susceptibility, a significant number of transitional C–H products may be destroyed and turn to expansive gels, which drives a significant strength reduction [44,55,94].

However, there are no data available on the ASR susceptibility of coarse WG aggregate (>5 mm size). Coarse WG aggregate increases the volume of the concrete and reduces the unit weight, but the porosity in concrete increases due to trapped micro-voids [65,89]. Due to the high porosity in concrete and low strength of WG aggregates, WGC suffers a major strength reduction with increasing amount of WG. Coarse WG can cause strength reduction in concrete due to the low adhesion between the smooth glass surface and cement paste and the lower strength of WG than that of stone aggregates [32,40,95]. Some typical observations, such as the decrease in concrete strength with the increasing amount and size of WG, are listed in Table 4. Therefore, it is necessary to follow the pozzolanic reactivity and ASR guidelines to select the particle size and amount of WG.

## 4. Current Challenges and Future Potential of WGC

### 4.1. Alkali–Silica Reaction in WGC

**Figure 15:**
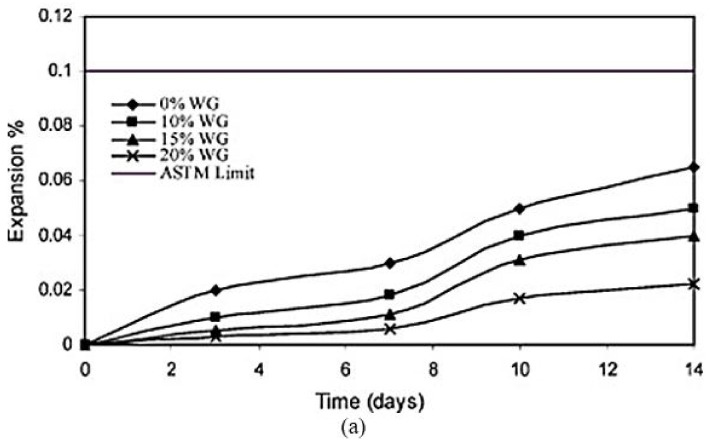

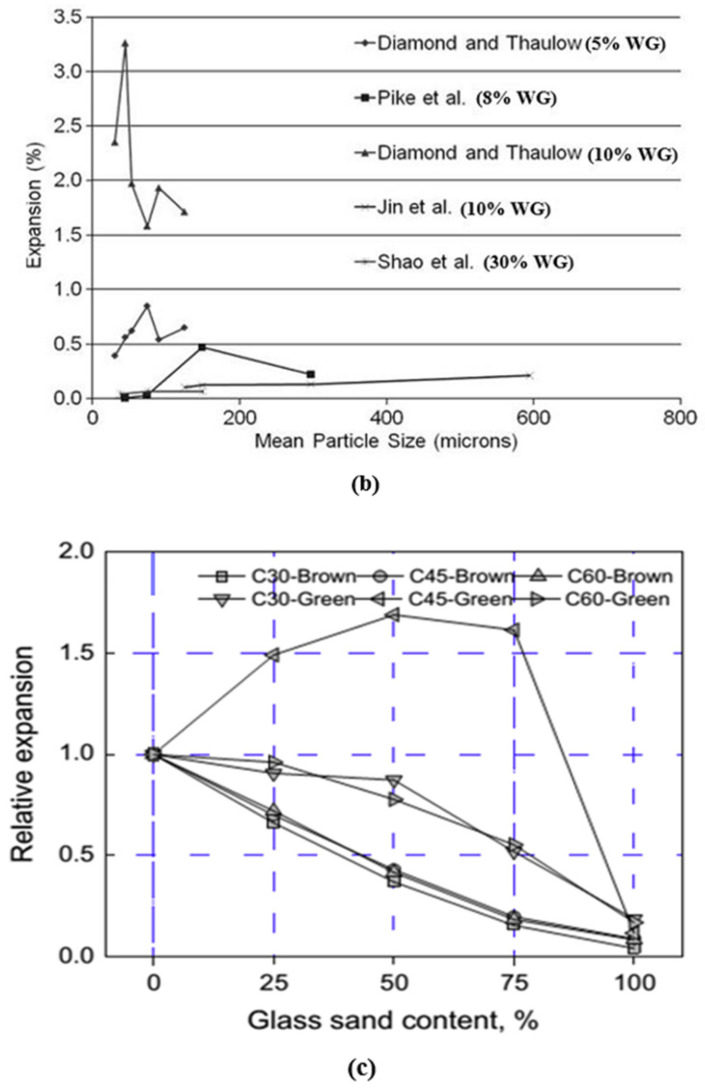
ASR expansion of WG concrete. (**a**) Expansion of mortar bars with WG (particle size: 0.6–2.36 mm) [51]; (**b**) variation in the ASR expansion with mean WG powder particle size [38]; (**c**) relative ASR expansion with varying glass content, color, and concrete grades [102].

The alkali content and silica in the WG powder react and form ASR gels. These gels are expansive and worsen when absorbing water or chemicals. Therefore, ASR of WG harms the strength and durability of concrete [1,6,97]. The ASR gel reacts with the hydration products (CH and C–S–H) and deteriorates the microstructure of concrete. Expanded ASR gels produce stress and create microcracks within the concrete core, which consequently leads to bond loss. The ASR vulnerability increases with the increasing amount of WG and its particle size; however, there are debates on the contribution of WG binder and WG aggregates to ASR expansion. Ismail et al. [51] observed decreasing ASR expansion in mortar with an increasing WG amount (particle size 0.6–2.36 mm) as a replacement for 0–20% fine aggregates (Figure 15a). In this case, the expansions in WGC were well below the ASTM limit (0.1%) [98]. However, [44] observed that when OPC was replaced by 30% WG (38 µm), the ASR expansion increased by 40% compared to the control mortar bar at only 16 days of age. Therefore, the testing condition and concrete mix proportion play an important role in ASR expansion in WGC.

Since ASR expansion increases with the particle size of WG, keeping the WG particle size below the critical value of 600–1000 µm is recommended (Table 5) [44,55,94]. Typically, there are more microcracks and voids inside coarser WG compared to finer ones, and ASR expansion occurs inside intrinsic microcracks and voids within WG particles [96]. By reducing the WG particle size from 150 µm to 38 µm, the ASR expansion is reduced from 0.065% to 0.043% when 15% aggregate in concrete is replaced by Rajabipour et al. [61]. Shao et al. [44] found much lower ASR expansion when WG particles of 38 µm size were used compared to glass powder with particle sizes of 75 µm and 150 µm. There was no unfavorable ASR expansion when 70% fine aggregate was replaced by 36–50 µm sized WG particles [56]. Therefore, finer WG particles are more resistant to ASR expansion, either as SCM or aggregate. A graphical representation of the impact of WG particle size on ASR expansion was presented by Federico and Chidiac [38] (Figure 15b), based on the results of previous studies [44,99,100,101]. The graph (Figure 15b) illustrates that the studies reported different ASR expansions due to the variation in compositions and testing conditions. Coarse WG particles show low reactivity at the early age of curing, thus forming more ASR gels at a later age. Therefore, the fineness and amount of WG are not the sole factors in ASR expansion.

Regardless of the amount and particle size, the type and chemical composition of WG and the concrete mix proportions can also affect the ASR. For example, binary glasses (sodium silicate) cause more ASR expansion than soda-lime glasses due to the higher alkali released from binary glasses [38,41]. Brown glass shows better ASR resistance compared with green glass (Figure 15c) [102]. Dhir et al. [103] reported that green glass showed more expansion of mortar bars compared to clear glass. On the contrary, Zhu et al. [94] reported that due to the presence of Cr ions, green glass possesses better alkali resistance compared with clear and brown glasses. The presence of Pb, K, Na, Pb, and B in the raw materials (which come from glass modifiers) accelerates the entire ASR gel formation and may also cause leaching [78,97]. Further, the presence of stabilizers (CaO and MgO) and lithium ions suppresses the expansion by changing the ASR gel composition [69,104]. There are debates about the reactivity and ASR expansion of glasses due to color because glasses of the same color could possess different chemical compositions. The effect of particle size on ASR expansion for green and brown glasses is also unclear due to contradictions among previous studies [10,95]. The variation in the ASR of glass due to the disparity in the chemical composition of glass and the proportion of concrete mix (with WG) needs considerable attention to draw a conclusion, as the impact of these parameters on ASR is still unclear, and the reasons behind these have not been clarified.

**Table 5:** ASR expansion of WG-based cementitious composite.

WG Type	Replacement Condition	Particle Size of WG	Age of Testing (days)	ASR Observation Compared to Control Specimen	Ref.
Soda lime glass	25% cement	10–20 µm	14	6.25% increased expansion	[97]
Fluorescent lamps	30% cement	38 µm	16	46.3% reduced expansion	[44]
Mixed WG	25% cement	≤100 µm	42	27.4% decreased expansion	[75]
Bottle glass	15% fine aggregate	<5 mm	14	20% increased expansion	[73]
Cathode ray tube glass	10% fine aggregate	<4.75 mm	14	11.5% increased expansion	[105]
Mixed colored glass	5% fine aggregate	<475 mm	14	14.5% increased expansion	[106]

### 4.2. Current Research Gap and Future Potential of WGC

**Figure 16:**
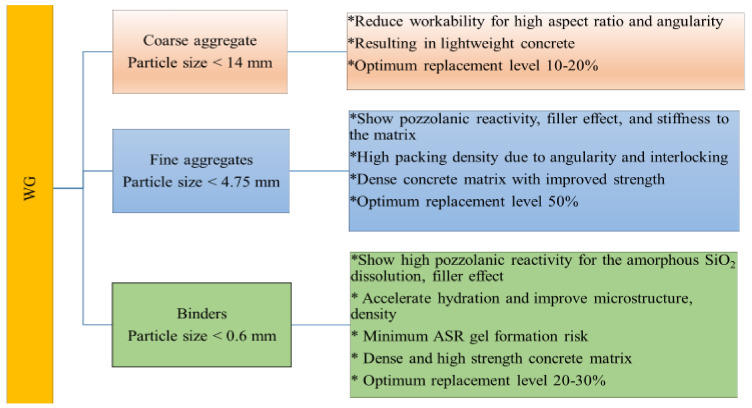
Performance and optimum replacement level of WG in cement concrete according to particle size and role [4].

Based on current practice, up to 30% cement, 50% fine aggregate, and 20% coarse aggregate in concrete can be replaced by WG of specified particle size without compromising the strength and grade of concrete (Figure 16) [65,69,78]. These WGC are being used to develop concrete-paving blocks due to their high abrasion resistance, high resistance to drying shrinkage, and water absorption [31,92,107]. The improved density and reduced porosity of WG powder-based concrete satisfy the criteria for high durability pavement materials [108]. WG can also be used to develop self-compacting concretes, self-healing concrete, and engineered cementitious composites as a replacement for fine aggregate and binder [47]. Several beneficial applications of WG concrete have been revealed, but their long-term mechanical performance has not been investigated. Moreover, there are no data available on the impact and fatigue resistance of WG concrete. The serviceability of WG concrete in terms of a reduction in carbon emissions and sustainability has not been investigated previously. Few studies have considered the application compatibility of WGC in reinforced concrete members, and their designs and performances have not been evaluated. A proper justification for performance is required to increase the application of WG-based cement concrete in real structures.

Although several advantageous uses of WG as a construction material are noted, the ASR challenges and bond strength limitations are critical issues for the future development of WGC [109]. Considering a well-graded WG powder, uniform silica dissolution can be ensured to improve the pozzolanic reactivity and reduce the ASR during the hydration stage and hereafter. There are contradictions about the effect of the color of WG on the ASR expansion of WGC, which need to be clarified through future research. Pre-treatment of WG using heat treatment, immersion in NaOH or Ca(OH)_2_ solution showed positive influences on the ASR reduction and strength in WGC [110].

The reaction products formed along the surface of WG during immersion in Ca(OH)_2_ solution enhance the pozzolanic reactivity of WG, and high bond strength concrete is developed. However, the chemical changes and optimum treatment conditions have not been identified, and the sustainability of this treatment process is not discussed in the literature. Studies on the addition of fibers, low-cost or recycled waste polymers, and proper milling processes to enhance the bond strength within WG concrete are lacking. Further, guidelines on the comparative and correlative descriptions of the internal chemistry, microstructure, strength, and serviceability properties in WG-incorporated OPC concretes need to be established through future research.

## 5. Glass as Precursors in Geopolymer Concrete

### 5.1. Geopolymerization of Glass Powder

#### 5.1.1. Chemical Role of WG and Other Precursors

**Figure 17:**
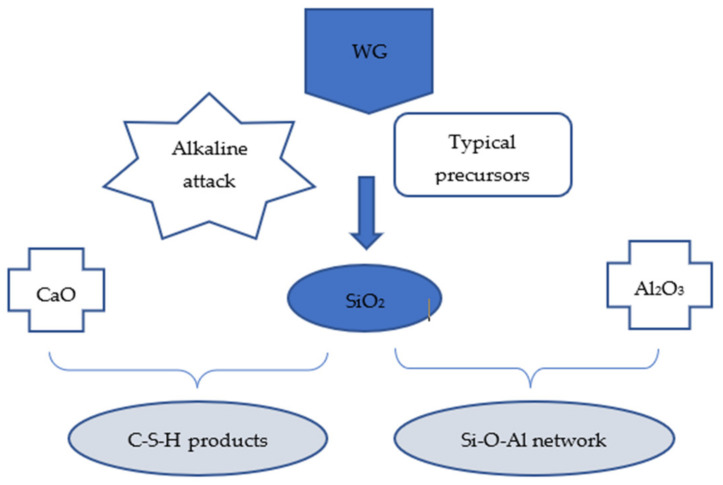
Different reaction product formations after alkali activation and geopolymerization of WG and other typical precursors.

Geopolymer formation starts from the dissolution of silica and alumina out of the precursors, followed by their gelation, reorganization, and polymerization into geopolymer binders [111]. As a precursor, WG mainly releases SiO_2_ and a small amount of Al_2_O_3_ and CaO depending upon its chemical composition to contribute to the geopolymerization process [109,112,113]. The hydration products in glass-based geopolymers are different from those in high-Al and low-Ca geopolymer concretes (Figure 17) [114]. Gels with low Al and Ca content and high SiO_2_ content are formed after the complete geopolymerization of alkali-activated glass-based precursors, regardless of the curing condition [115]. However, for a high-calcium system, such as slag-based geopolymer concrete, the hydration products are generally C–S–H gels. Fly ash and calcined clay-based geopolymer concrete with WG can be regarded as a low-Ca system in which the main hydration products are N-A-S-H type gels with low Ca and high Al content [113,116]. For high-Ca fly ash- and slag-based systems, the dominant hydration products can be C-(N)-A-S-H gels [64], whereas the elemental chemistry of the solution favors the formation of SiO_2_-rich gels in a composite paste of alkali-activated WG and other precursors [33,109]. However, the impact of the other ingredients in WG on hydration products and the type of gels has remained unrevealed in the literature.

#### 5.1.2. Parameters for Geopolymer Concrete with WG Precursor

**Figure 18:**
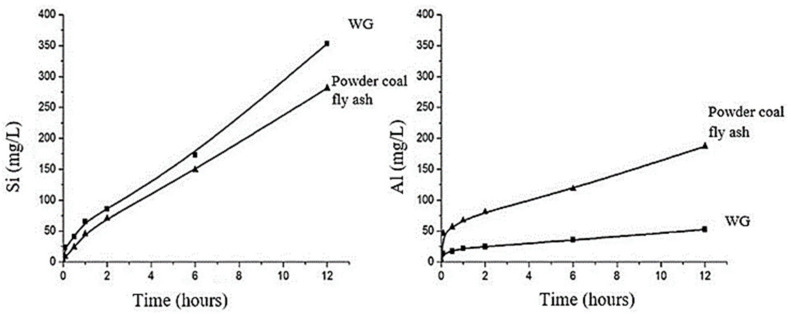
Si and Al dissolution from WG powder and fly ash [64].

Silica dissolution from WG precursors is dependent on the fineness and amount of WG, concentration, type of activator, mixing, and curing environment of the geopolymer mix with WG. Finer WG precursors (particle size < 75 µm) release more SiO_2_ and Al_2_O_3_ during geopolymerization than coarser ones and result in good geopolymerization regardless of other chemical factors [116,117]. Zhang et al. [64] compared the dissolution rates of Si and Al from WG and fly ash. As shown in Figure 18, at 20 °C, the silica dissolution rate from WG powder (50% finer than 5.07 µm) is much higher than that of powdered coal fly ash (50% finer than 33.19 µm), which is generally used as the precursors for geopolymer. Additionally, with the increasing amount of WG, the silica dissolution increases, which consequently increases the Si to Al ratio significantly. A moderately high Si/Al ratio is required to form Si–O–Si bond, but with a low Si/Al ratio, Si–O–Al and Al–O–Al bonds are formed [118,119]. Zeolite products are formed during geopolymerization when the Si/Al ratio is too high. WG does not release enough alumina, although both Al and Si are needed for geopolymerization (Figure 18). However, when WG is beyond the optimum level (30% of the precursor), unreacted silica will remain in the matrix due to the insufficient amount of Al to react with Si. Khale and Chaudhary [120] recommended a Si/Al ratio in the range of 3.3–4.5. Therefore, to keep the Si/Al ratio within the right range, more than 20–30% of WG is not suitable as a precursor.

**Figure 19:**
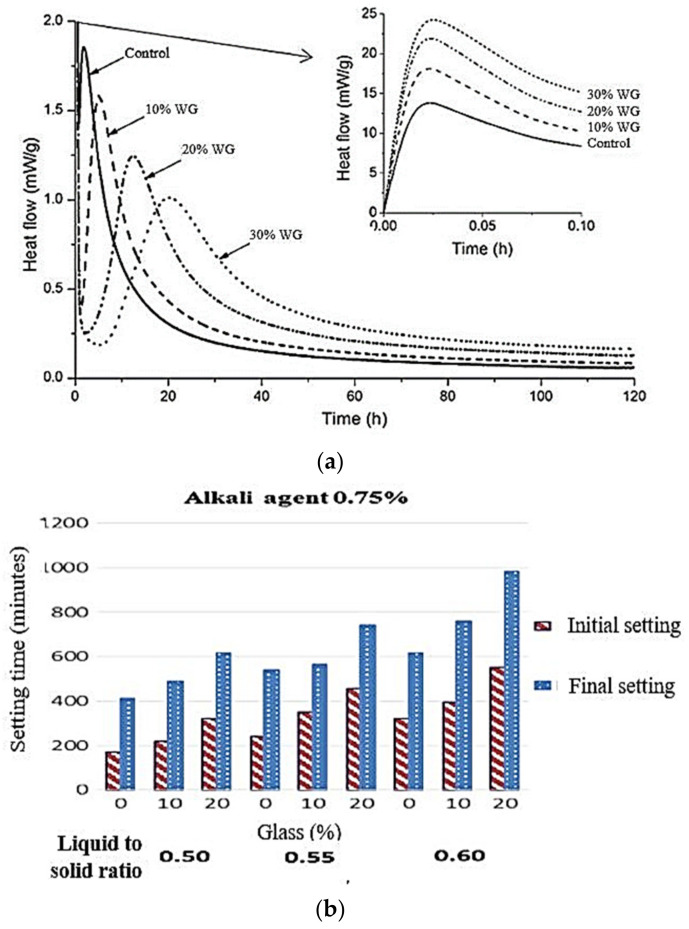
Hydration characteristics of WG powder-based geopolymer concrete. (**a**) Heat flow rate of WG powder-based geopolymer concretes [64] (**b**) Setting time variation in WG powder-based geopolymer concretes [67].

Moreover, WG prolongs the setting time of geopolymers. Regardless of the activator concentration, a prolonged setting time was observed with an increased amount of WG in the binder. The setting time generally correlates with the second peak of heat flow during hydration. A comparison of Figure 19a,b indicates that when the amount of WG increases, the second peak of heat flow is lowered, and its formation is delayed. As pointed out by Liu et al. [116], the presence of alkali atoms reduces the polymerization process when WG powder is included in geopolymer formation. Y. Liu et al. [116] reported that the proportions of CaO and Fe_2_O_3_ in raw materials should be below 15–20% to obtain high-strength geopolymer concrete. Further investigation is needed to clarify the role of WG in early-age hydration products and the chemistry of the gels in alkali-activated WG.

A high concentration of alkaline activator is required to ensure the high dissolution of silica from WG [121]. However, excessive alkaline media can cause unnecessary delays in the decomposition of aluminosilicate products and geopolymerization due to minimal ion mobility [119,122]. The suitable concentration range for an alkaline activator is around 8–10 M, depending on the type of activator, liquid-to-solid ratio, and the proportion of the precursors [22,115]. Commonly adopted activators are NaOH, Na_2_SiO_3_, and their combinations [23,117]. Given that the WG powder is pozzolanic, CaO can be an alternative activator for WG powder-based geopolymers [52]. The addition of a CaO activator imparts hydraulic properties in pure WG powder paste, which positively influences the strength and microstructure development of geopolymer [123]. Some other effective activators for WG-based geopolymers are KOH, Na_2_SO_4_, and Ca(OH)_2_ [115,116,124,125]. However, according to Torres-Carrasco and Puertas [115], the effect of the activator type and its concentration on the composition of the final geopolymer product is insignificant, but their impact on strength development is considerable. The effectiveness of each activator depends on its concentration, temperature, pH, and curing conditions; this effect should be considered before adopting activators in the production of WG-based geopolymer concrete for structural purposes.

Moreover, mixing and curing temperatures can remarkably affect the geopolymerization reaction and the performance of the concrete. Toniolo et al. [126] claimed that the glass powder mix should be allowed for around 6 h at 80 °C to release a high amount of silica into the solution. The WG powder releases some alkali content but does not dissolve sufficient silica at room temperature; thus, raised-temperature curing is required [29,126,127]. However, mixing at room temperature but curing at a raised temperature (40–60 °C) is also effective to dissolve sufficient SiO_2_ and Al_2_O_3_ from WG to complete the geopolymerization process [29,109,117]. On the contrary, Arulrajah et al. [108] reported that the strength and stiffness of fly ash geopolymer concrete cured at 50 °C are comparable to those of the 50% WG-added geopolymer at 21 °C curing. Therefore, curing at room temperature using other reactors could be efficient, as mixing and curing at high temperatures is complicated and costlier for large volume geopolymer casting. High temperatures may, however, result in enlarged pore size in hardened geopolymer concretes [128]. Therefore, these contradictions and disparity should be cleared by further investigations of the behavior of the WG precursor and the impact of its ingredients on geopolymerization. These investigations will be helpful in developing guidelines for mix design, curing conditions, and optimum level of the WG for sustainable and high-performance geopolymer concrete.

### 5.2. Properties of Geopolymer Concrete with Waste Glass Precursors

#### 5.2.1. Physical Properties

##### Workability of Geopolymer Concrete with WG Precursor

**Figure 20:**
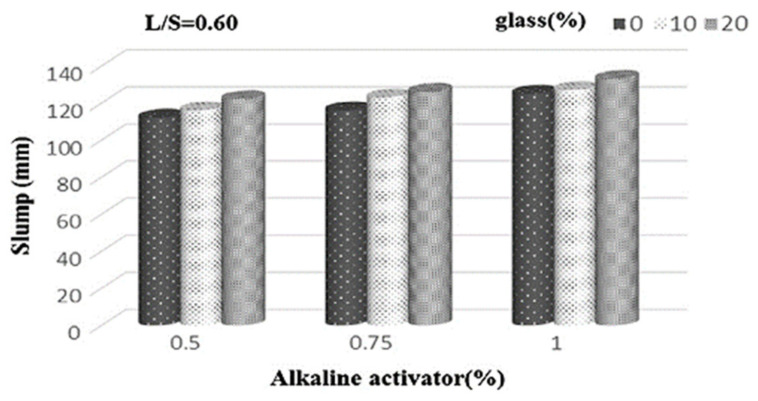
The slump of geopolymer concretes with varying WG powder (fineness = 2009 cm^2^/g) and activator content [67] (L/s—liquid to solid ratio; WG = 0–20%).

The workability of alkali-activated geopolymer concrete increases with the increasing content of WG powder (Figure 20). About 10–20% WG (fineness 2009 cm^2^/g) was used by Wang et al. [67] to replace slag precursor, resulting in an increasing trend in slump value with increasing WG amount. Wang et al. [129] reported a 34.4% increase in slump after adding 40% glass powder (particle size < 600 µm) in slag-based alkali-activated mortar. This was attributed to the negligible water absorption by WG, and its smooth surface made the geopolymer mix more flowable. The liquid-to-solid ratio and the concentration of activator are essential parameters that control the workability of geopolymer [66,109]. However, the variation in the flowability of geopolymer concretes with the particle size and roughness of WG powder has not been studied in the literature. Like cement concrete, these parameters should have an impact on slump values, which should be investigated.

##### Density and Microstructure of Geopolymer Concrete with WG Precursor

**Figure 21:**
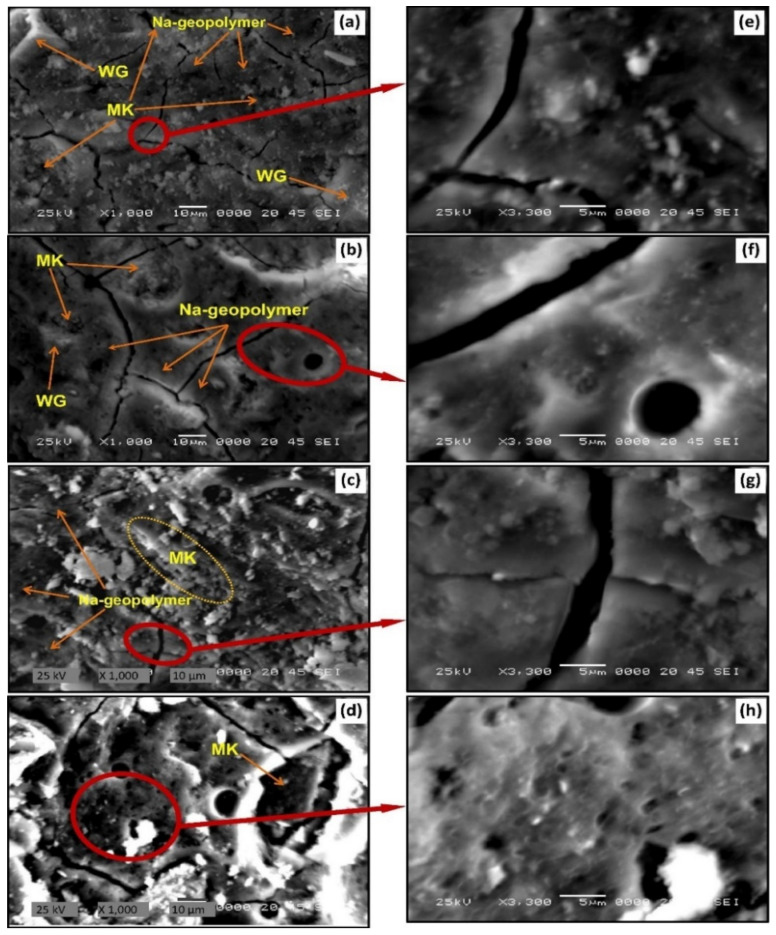
SEM micrographs (1000×; 10 μm) of WG/MK-based geopolymers cured at (**a**) 25, (**b**) 40, (**c**) 60, and (**d**) 80 °C. Red circles in (**a**–**d**) are magnified (3300×; 10 μm) into (**e**–**h**), respectively [118].

In geopolymer concrete, WG accelerates the geopolymerization reaction and acts as a filler when used for the replacement of slag or fly ash. Therefore, the bond strength within the WG geopolymer network is high, consequently leading to a dense matrix formation [29,128]. The microstructure of the geopolymer matrix depends on the main precursor characteristics and the replacement level with WG. Metakaolin (MK) is a SiO_2_- and Al_2_O_3_-rich precursor. When metakaolin is replaced by a finely ground WG (3%), which is SiO_2_ rich and contains a low amount of CaO, a Na-aluminosilicate amorphous matrix is formed at room temperature (Figure 21) [118]. The fractured and porous microstructure in the room temperature cured geopolymer matrix turned to a homogenously and closely packed dense matrix when cured at a raised temperature of around 60 °C (Figure 21). With the raised curing temperature, the unreacted particles were also reduced in number, which is consistent with the geopolymerization behavior of the WG.

However, the density of the geopolymer decreases with the addition of excessive amounts and coarse-sized WG precursors because the agglomeration of the WG powder delays and hampers the geopolymerization process [57,112]. The current guidelines are not sufficient for designing the optimum particle size, liquid-to-solid ratio, activator concentration, curing condition, and replacement level of WG-precursor geopolymer. Therefore, comparative studies are required in this field to establish a mixed design for WG-geopolymer concrete.

#### 5.2.2. Mechanical Properties of Geopolymer Concrete with WG Precursor

**Figure 22:**
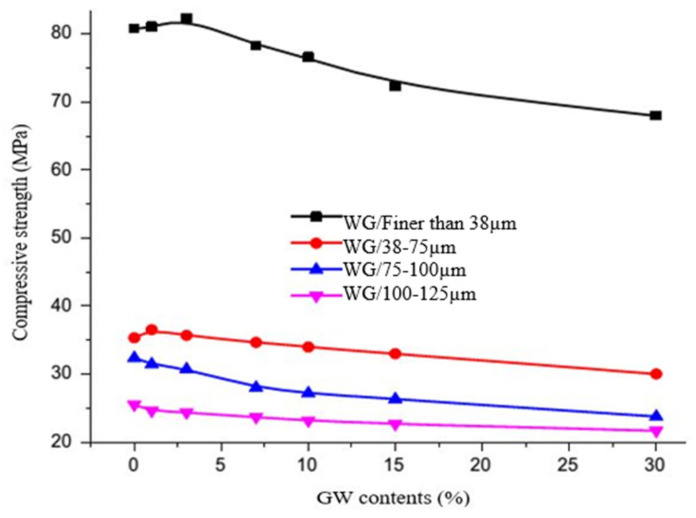
Effect of WG powder precursor on the compressive strength of geopolymer [118].

The mechanical properties of geopolymer concrete depend on the geopolymerization process, which depends on the dissolution of elements (silica and alumina) from precursors. WG effectively participates in the geopolymerization process by releasing silica in high alkali-activating media and improves the strength of geopolymer concrete, depending on the type and content of WG and its activation methods [24,29,130]. A typical variation in the compressive strength of geopolymer with varying particle sizes and WG precursor content as a replacement for metakaolin is shown in Figure 22 [118]. This figure indicates that the addition of finer WG powder can yield a higher strength in the geopolymer due to the higher dissolution of SiO_2_ compared with the coarser WG powder. The most effective particle size for the WG precursor was found to be 38 µm and finer [44]. The strength of geopolymer concrete can increase with the amount of WG precursors. El-Naggar et al. [118] observed 2% higher compressive strength in geopolymers after 28 days of curing when they replaced 3% metakaolin precursor with WG powder (<75 µm). Novais et al. [119] demonstrated that the replacement of metakaolin with 12.5% WG powder (<75 µm) resulted in 46% improved compressive strength in geopolymer concretes. The strength development rate of geopolymer increases with age due to the high dissolution of silica over time [29,119]. However, WG precursors beyond 30–50% are ineffective for successful geopolymerization due to the deficiency of Al_2_O_3_ and the presence of unreacted glass particles in the composite [119,128].

**Figure 23:**
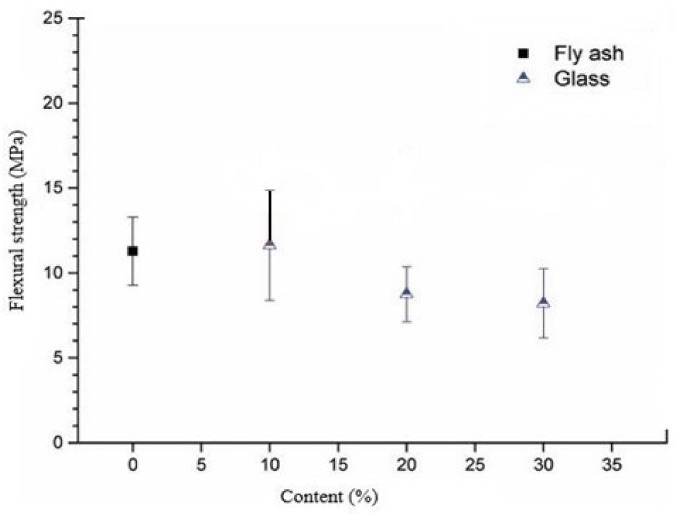
Flexural strength of geopolymer concrete with WG (particles size ≤ 60 µm) compared to the fly ash precursors [131].

Figure 23 represents the observation from the literature [131], which describes that the flexural performance of fly ash geopolymer concrete is not satisfactory when the amount of the WG powder exceeds 10%. Flexural strength of concrete increases with the addition of WG (10–20%) because WG has high stiffness and interlocking due to its angularity [132]. The load-deflection behavior of geopolymer concrete with WG under the bending test is linear, and the failure pattern is the brittle type [131]. The slope of the load-deflection curve varies due to discrepancies in the elastic modulus [131].

There is a close relationship among the activator’s properties, Si/Al dissolution from precursors, and geopolymerization. Sethi et al. [133] found a 178% improvement in compressive strength at the 7-day curing age of geopolymer concrete, where a 4 M NaOH activator was used to activate the precursor mix of 5% WG, 25% slag, and 70% fly ash. With the increasing concentration (2–12 M) of activator solution, the strength of the geopolymer increases, but beyond the optimum level (8–12 M), it showed a negative effect [130]. Initially, activator of higher molarity helps to dissolve more elements (SiO_2_, Al_2_O_3_, and CaO) from precursors (WG, fly ash, or others) [4,130]. On the other hand, excessive alkalinity creates high-rate precipitation of silica and agglomeration of ions, which makes the geopolymer composite weak and porous.

**Figure 24:**
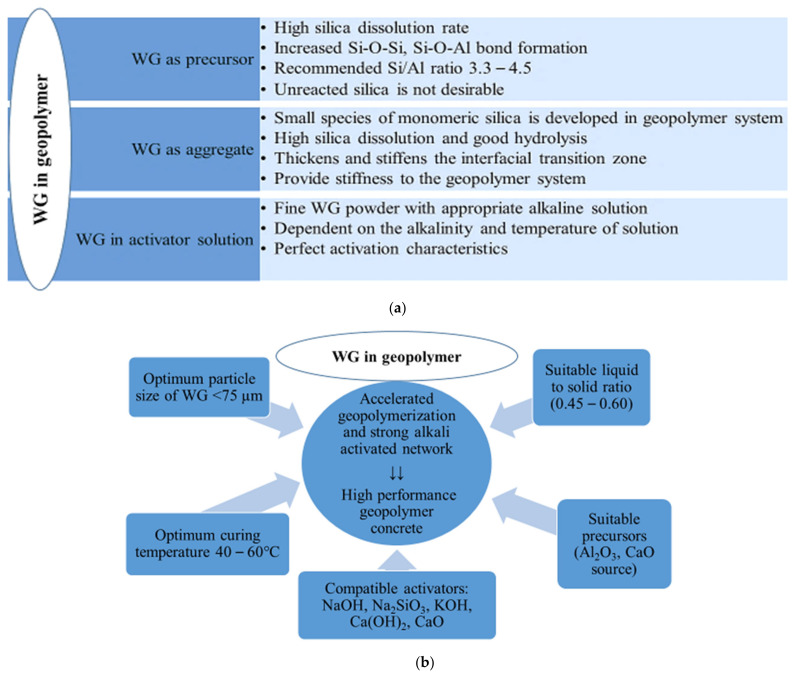
Different roles of WG and the impacts of different parameters on the performance of geopolymer concrete [4]. (**a**) Reactivity and roles of WG in geopolymer concrete; (**b**) Crucial process parameters in alkali activation and geopolymerization with WG.

The investigations of the mechanical properties of WG geopolymer concretes are listed in Table 6, and the roles, reactivity, and process parameters of WG-based geopolymer concrete are listed in Figure 24a,b. This review reveals that the WG precursor improves the mechanical strength of geopolymer. The optimum level of geopolymer precursor replacement with WG powder is approximately 20–30%, which may vary with particle size and mixing conditions. However, this observation also reveals a lack of deep investigation into the mechanical and chemical analysis of geopolymer concrete with WG. The chemical characteristics and strength development stages of WG precursors have not been fully revealed in previous research on geopolymer concrete with WG.

## 6. Glass as an Aggregate in Geopolymer Concrete

### 6.1. Role of Glass Aggregate

Fine aggregates create a distinct ITZ and increase the volume of geopolymer concrete (Figure 24). The fine WG aggregates provide stiffness to the geopolymer matrix, enhance geopolymerization, produce a higher amount of high-strength Si–O–Si links, and therefore improve the microstructure and strength of geopolymer concrete [23,29,30,118,119]. The dissolution of silica from the WG aggregate and precursor are dependent on the same factors. However, the particle size of WG aggregates is coarser than the precursors. The surface of the WG aggregates can react with the precursor and activator and create a strong interfacial bond [35]. The Si–O– bond formation and heat flow during hydration of WG-fine aggregates are evident in the literature [29]. The inclusion of fine WG powder as an aggregate in geopolymer concrete reduces heat flow during hydration due to the presence of highly alkaline media [29]. Zhang et al. [64] observed double peaks in the heat-flow curve (Figure 19a) for the hydration of WG. The first peak represented the saturation and dissolution of mixing particles into an activator solution, and the second wider peak was related to heat production due to the formation of the reaction products. The hydroxyl ions attacked the Si–O–Si bond and increased the silica dissolution rate.

However, to develop high-strength bonding, the presence of many silanol groups in the solution is needed, along with a high silica dissolution [35]. Thus, monomeric and small species silica accelerate the hydrolysis reactions during hydration and enhance geopolymerization. Puertas and Torres-Carrasco [127] reported that a high pH (approximately 13.6) is needed for the favorable dissolution of silica from WG powder in monomeric form. In a WG-added fly ash geopolymer system, small species of silica form around WG aggregates due to the presence of high-alkaline media, and these silanol groups can accelerate the fly ash dissolution and geopolymerization process [35]. By contrast, in a sand-based geopolymer concrete system, a large species of the silica-rich network is formed, which may hinder the dissolution of precursor and rate of geopolymerization [35]. The WG aggregates act as fillers due to their fineness and enhance interlocking due to their rough texture [47], which yields high bond strength in the geopolymer system with WG aggregates. As reported in the literature [29,66], WG-based geopolymer concretes are generally made of finer aggregate (<5 mm) particles than the OPC concrete. However, for mass construction, coarse aggregates are suitable for increasing the volume of the geopolymer concrete. Although no previous study has accounted for coarse WG aggregates in geopolymer construction, investigations are needed to analyze the behavior of coarse WG in geopolymers.

### 6.2. Properties of Geopolymer Concrete with Waste Glass Aggregates

**Figure 25:**
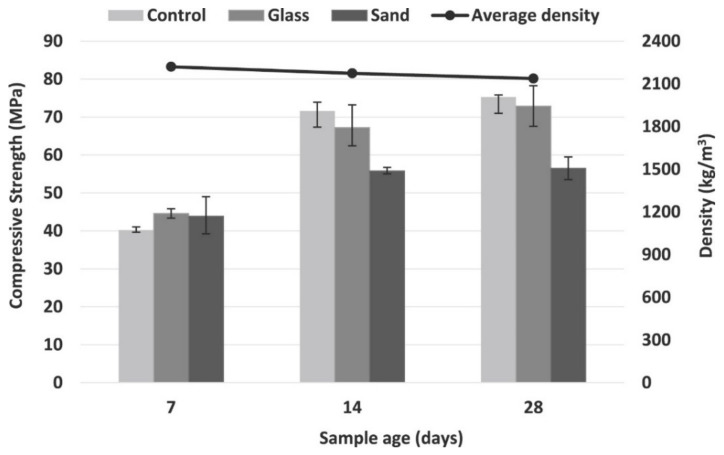
Density of geopolymer concretes with different aggregates at different ages [35].

The density of geopolymer concrete increased with the addition of WG aggregate. Figure 25. exemplifies that the addition of 25% of WG (particles of 2–5 mm size) in the replacement of natural sand results in a denser matrix of fly-ash-blended geopolymer concrete. The control geopolymer was cast without any aggregates. The high alkalinity of the WG-added geopolymer system and high rate of silica dissolution led to the formation of small monomeric silica species [35]. The monomeric silica can accelerate the hydrolysis reactions during hydration, enhance geopolymerization, and consequently compact density [35]. Hajimohammadi et al. [23] added 30% WG aggregate (0.4–2 mm) with 70% geopolymer binder and found a 77% improved strength in geopolymer foam concrete compared to the geopolymer concrete with sand aggregate. The authors reported that the geopolymer with WG aggregate was 25% stronger than the geopolymer with sand aggregate. The surfaces of WG aggregates interact with the precursor paste and form a strong bond when activated by an alkaline solution. The unreacted content of silica can also act as a reinforcement and microaggregates to fill the fine pores within geopolymer concretes. However, WG content must be within the optimum limit (around 30–50%) because excessive unreacted silica in geopolymer can cause porous geometry and consequently reduce the density and strength of the final composite [22].

## 7. Role and Impact of WG as an Activator

**Figure 26:**
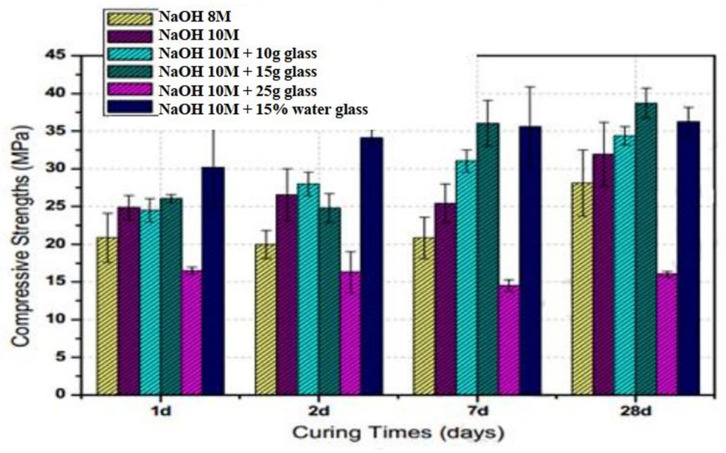
Effect of WG (particle size < 45 µm) solution as an activator in fly ash-based geopolymer concretes [130].

WG-derived solutions can be effectively used as activators for geopolymer concrete. The WG releases a significant amount of alkalis and silica in NaOH and water solution when being heated, and the heat-treated solution meets the requirement of a water glass activator [124,134]. Sodium silicate activator, which is produced by heating the WG powder in water and NaOH mix, is suitable for use as an activator for fly ash blended geopolymer. The solution derived from the heat treatment of WG powder with a NaOH/Na_2_CO_3_ solution can be effectively used as an activator for slag-based geopolymer concretes. For example, 10 M NaOH with 10–25 g of WG powder solution was used as an activator in a prior study by Torres-Carrasco and Puertas [130] (Figure 26).

Torres-Carrasco and Puertas [130] reported the successful activation of fly ash with a WG-derived activator because of the high Si content in WG. The particle size of WG powder, preparation method, temperature, and the characteristics of alkaline media were the most important parameters that control the successful production of glass-based activators. A high synthesis temperature (>330 °C) was required to develop a high amount of activator from WG [134]. However, the WG activator, synthesized at 150 °C, was more effective than other activators synthesized at a higher temperature (in terms of the geopolymer strength). A high amount of unreacted silica and flash setting of geopolymer could occur for high-temperature synthesis and activation; consequently, the strength may decrease. However, there are very limited studies on the preparation of activator solutions from WG. Therefore, further studies on the preparation process and properties of the WG-derived activators are required to have the proper knowledge and concluding ideas on this topic.

## 8. Current Challenges and the Future Potential of WG Geopolymer Concrete

### 8.1. Alkali–Silica Reaction due to WG in the Geopolymer

**Figure 27:**
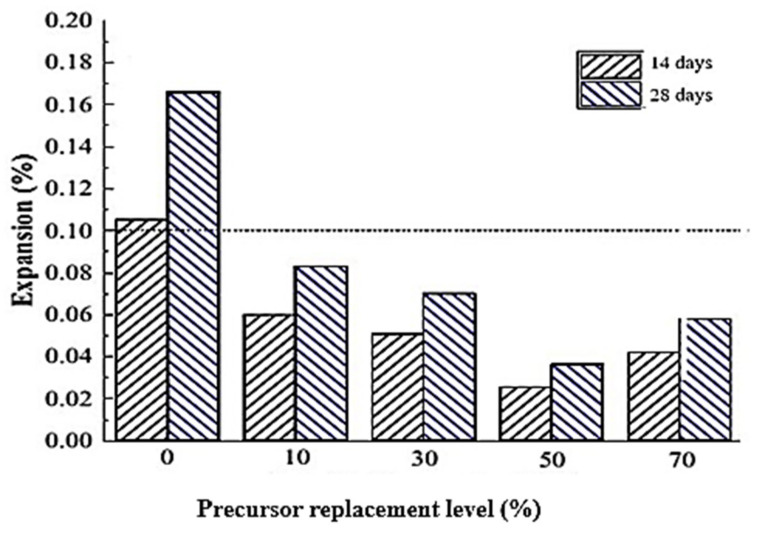
ASR expansion in alkali-activated geopolymer concretes with WG powder (particle size < 32.86 µm) [49].

Geopolymer concretes are alkali-activated; thus, the risk of ASR expansion is high since the reactivity of the WG increases at high pH [79]. Nonetheless, as observed by Williamson and Juenger [135], the ASR expansion was only 0.02% in geopolymer concrete and 5% in OPC concrete after 24 months of exposure time. The authors reported that the use of highly reactive aggregates and high-concentration activators beyond the optimum level are the leading causes of ASR expansion in geopolymer concrete. However, in most cases, ASR gel formation in geopolymers is much lower than in OPC concrete [49]. This condition could also be attributed to the flat rate of silica dissolution from the WG powder in a geopolymer at an early age of activation. The subsequent contact of alkaline solution could increase the silica dissolution rate at a later age and accelerate geopolymerization [135]. Strong sodium silica gel is formed in geopolymer concretes over time through alkali reaction, which contributes to minimizing ASR expansion by balancing many alkali cations [29,123]. However, the ASR expansion can increase with the increasing amount of WG and the age of geopolymer concrete. Mortars show increasing resistance to ASR expansion with increasing WG powder (particle size < 32.86 µm) up to 50% replacement of slag (Figure 27) [49]. Further addition of WG powder caused an increasing trend in ASR expansion, although it was still below the expansion caused in geopolymer without WG. The increasing expansion above 50% replacement may be caused by the agglomeration of unreacted silica and delayed geopolymerization, which makes the alkali contents available for expansive ASR gel formation. Therefore, concerns about the ASR expansion effects in geopolymer concretes caused by the addition of WG cannot be neglected. However, there is no direct relationship observed in the literature between the molarity of activator, liquid-to-solid ratio, and ASR expansion of WG-based geopolymer concrete; therefore, studies are required to relate these parameters to WG-based geopolymers.

### 8.2. Current Research Gap and Future Research Potential

**Figure 28:**
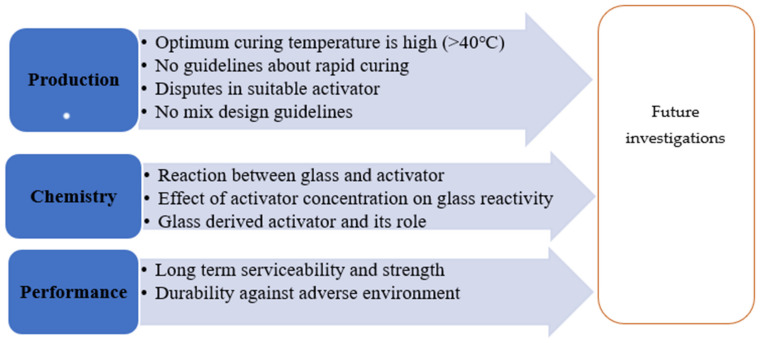
Current research gaps and future research questions in the production, chemistry, and performance of WG-based geopolymer concrete.

As an abundant source of silica, WG is suitable for use in alkali-activated autoclaved aerated concrete, foamed concrete, geopolymer foams, and concrete [46]. The WG particles improve the thermal insulation and serviceability properties of autoclaved aerated, foamed, and geopolymer concrete [22,46]. WG precursor can be effectively adopted to develop road-subgrade geopolymer material [108]. WG-based geopolymers can be used to develop low-carbon emission masonry units [112]. Therefore, there are significant scopes for using WG in geopolymer composites. However, there are very limited studies available on the properties and performance of WG-based geopolymer concrete, which is a barrier to extending the application of WG geopolymers. Therefore, investigations considering the variation of activator concentration, WG particle size, and liquid to solid ratio and their effect on the mechanical properties of WG geopolymers, especially splitting tensile strength, flexural strength, and modulus of elasticity, are needed (Figure 28).

Notably, most of the studies on geopolymer concrete have not considered the use of coarse aggregates, which is important for high-volume casting. The addition of coarse aggregates (conventional and WG) enhances the thermal, fire, and elastic properties of geopolymer concrete. Further, the optimum curing condition for WG-based geopolymer concrete needs to be standardized because current knowledge is not sufficient, and raised temperature or oven curing is not a sustainable solution. There is potential for developing WG activators for geopolymer concrete, but due to limited studies on this issue, no certain guidelines exist. Some additional challenges include the lack of guidelines and approval for the standards and the limited availability of admixtures and precursors that are compatible with WG in geopolymer concrete. These issues should be considered in future research, and guidelines should be established to widen the applicability of WGC in the construction sector.

## 9. Conclusions

This review includes a critical discussion of the current research progress and future potential of waste glass (WG) in concrete production. Glass can be effectively included in cement and geopolymer concrete, substituting for conventional raw ingredients. The chemical composition and physical characteristics of WG offer compatible performance within the concrete. Based on the reviewed literature, the following conclusions can be drawn:

- Glass powder acts as a source of pozzolanic silica for cement and geopolymer concrete. For best pozzolanic reactivity, the particle size of WG and optimum binder replacement level should be below 75 µm and 30%, respectively. Silica, calcium oxide, and the small amount of alumina in WG participate in the hydration reactions and accelerate the formation of hydration products. A high curing temperature of around 40–50 °C and a proper water-to-binder ratio (<0.5) are useful to increase the pozzolanicity and hydration of WG in concretes.
- Fine WG powder (particle size < 150 µm) acts as a filler and pozzolanic material, increases the density, and reduces the porosity of concrete composites. Consequently, the mechanical performance of concrete is reliably enhanced. The most suitable WG conditions for improving the pozzolanicity and mechanical performance of concretes are WG powder with particle sizes less than 75 µm and the optimum cement replacement level (20–30%). As fine aggregates, a replacement level of approximately 50% is feasible to yield optimum strength in WG concrete.
- The mechanical performance of geopolymer concrete with WG aggregates, precursors, and activators has been consistent. Silica dissolution takes place from the WG in an alkaline activator solution, which accelerates the geopolymerization process. The replacement of precursors in geopolymers has been widely investigated, and the optimum replacement level lies within 30–50% with a WG powder particle size less than 75 µm. The requirement of an alumina source is crucial for completing the geopolymerization of WG-based geopolymer concretes. However, the intermediate reaction products of calcium or sodium aluminosilicate hydrates also showed significant stability in WG-based geopolymer concrete.
- The highly reactive surface of fine WG aggregates reacts in activated media to create a high-strength geopolymer network. For sodium-silicate activator solution preparation, the particle size of WG powder could be reduced to under 45 µm for better dissolution of silica and alkalis from WG. For WG powder-based geopolymers, the solid-to-liquid ratio (0.5–0.6), additional alumina sources, curing temperature (50–60 °C), and alkalinity of activator (8–10 M) must be controlled to achieve optimum performance.
- The most critical issue of glass incorporation into concrete is the alkali–silica reaction and expansive gel formation within the composite. This issue is less critical for geopolymers than cement concrete, but it cannot be ignored. The microcracks in WG particles cause more ASR vulnerability. ASR expansion in concrete can be minimized by using an optimum level of around 10–30% fine WG powder (<75 µm) to replace cement rather than aggregates and by adding recommended by-products, such as silica fume, fly ash, and slag.

There are still some challenges related to the use of WG in cement and geopolymer concrete, but the current research outcomes have revealed the potential economic and environmental benefits of WG as a construction material. Future research should widen the application of WG to commercial fields. This review highlighted the lack of investigations on WG-based cement and geopolymer concrete, especially in the areas of critical exposure conditions and performance under short-duration vibration, impact, and fatigue loadings. Further investigations on these topics are required to develop widely applicable guidelines for the application of WGC in the construction sector.

## Figures and Tables

**Table 1:** Chemical composition of WG and OPC (LOI: loss on ignition).

Source or Type of WG	Chemical Constituents (Weight, %)											Ref.
	SiO_2_	Al_2_O_3_	Fe_2_O_3_	CaO	MgO	SO_3_	Na_2_O	K_2_O	B_2_O_3_	Other	LOI	
Glass bottles/soda-lime glass	71.40	2.54	0.37	11.2	1.6	0.16	12.25	0.36	-	0.04	0.82	[27]
	70.30	1.90	0.42	12.30	1.68	0.07	12.80	0.23		-	0.68	[30]
Windshield	75.15	0.95	0.31	8.95	2.34	0.36	-	0.64	-	-	11.30	[10]
Window/ tempered glass	72.21	1.087	0.174	8.93	3.63	-	14.38	-	-	0.57	-	[9]
Borosilicate	75.62	2.258	0.006	0.13	0.26	-	4.59	-	15.64	1.841	-	[9]
Cathode ray tube	54.86	9.88	-	2.98	1.27	0.1	3.87	2.36	-	24.52	0.16	[49]
OPC	21.73	3.60	1.5	63.20	3.20	2.5	0.96	0.27	-	0.03	1.90	[27]

**Table 2:** Summary of the mechanical properties of concrete with WG as SCM.

Replacement Level	WG Type	Specimen Properties (Cement: Fine Aggregate: Coarse Aggregate = C:FA:CA)	Variation in Mechanical Properties	Remarks	Ref.
5–30% cement	Mixed type (<100 µm)	C:FA:CA = 1:2.65:1.92 concrete, Water-to-cement ratio = w/c = 0.51	18% improved compressive strength for 5% WG powder Highest splitting tensile strength observed for 10% replacement level	Up to 20% cement replacement by WG powder results in high strength and durable concrete	[75]
10% of cement	Liquid Crystal Display (5 µm and 12 µm)	C:FA = 1:2.13 mortar, w/c = 0.4	Compressive strength improved by 22% and 11%	Finer WG powder yield more strength	[80]
20% of cement	Recycled fibrous glass (mean 8.4 µm)	C:FA:CA = 1:2.44:3.19 concrete, w/c = 0.5	24% higher compressive strength and flexural strength than the control specimens at 91 days of age	Silica dissolution makes surfaces of WG particles rough, and these topographical changes in the interface between WG and cement paste causes ASR	[85]
20–40% of cement	Glass bottle (<20 µm and <40 µm)	C:FA:CA = 1:2:1.89 concrete, w/c = 0.4	4.14% lower compressive strength than general cement concrete at 28 days of age with 20% WG of 20 µm particle size The WGC gains 54.08% more strength at 545 days of age compared to 28 days of age	Continuous evolution and refinement of the pore structure happens due to WG powder	[86]

**Table 3:** Chemical composition of natural river sand.

Source or Type of WG	Chemical Constituents (Weight, %)									LOI	Ref.
	SiO_2_	Al_2_O_3_	Fe_2_O_3_	CaO	MgO	SO_3_	Na_2_O	K_2_O	Other		
Glass	71.40	2.54	0.37	11.2	1.6	0.16	12.25	0.36	0.04	0.82	[27]
Natural river sand	78.6	2.55	2.47	7.11	0.46	-	0.42	0.64	-	7.6	[37]
Natural limestone aggregates	-	0.15	0.60	57.51	1.05	-	0.06	0.01	-	40.5	[10]

**Table 4:** Summary of the mechanical properties of WGC.

Replacement Level	WG Type	Specimen Properties (Cement: Fine Aggregate: Coarse Aggregate = C:FA:CA)	Variation in Mechanical Properties	Remarks	Ref.
0–100% fine aggregate	Flat glass and container glass (<4.75 mm)	C:FA:CA = 1:2:4 concrete, w/c = 0.5 with 20 MPa target strength for 28 days of age	• 10% and 4% improved compressive strength for 25% and 50% replacement levels, respectively, at 90 days of curing • A reduction in splitting tensile strength of concrete occurred	Weak ITZ was formed due to the low bonding between cement paste and glass aggregate	[84]
10–20% fine aggregates	Mixed type (0.6–2.36 mm)	C:FA:CA = 1:1.88:2.68 concrete, w/c = 0.53	• Flexural strength enhanced by 10.99% • Compressive strength improved by 4.23%, with 20% replacement level	For low replacement level, early age curing significantly contributed to strength development; for high volume replacement, a long curing period was required	[51]
10–60% coarse aggregates	Soda bottles (4–16 mm)	C:FA:CA = 1:1.85:3.2 concrete, w/c = 0.54	• 8%, 15%, 31%, and 49% decrease in compressive strength observed for replacement level 15%, 30%, 45%, and 60% respectively	Low adhesion of WG aggregate with cement paste is resulting low strength and highly brittle concrete	[1]
30% coarse aggregate	White glass (5–10 mm)	C:FA:CA = 1:1.75:2.75 concrete, w/c = 0.32	• 40.72% increased compressive strength	To reduce ASR and increase strength of WGC, a low w/c ratio with workability admixture is recommended	[96]

**Table 6:** Observation of the strength of WG-based geopolymer concretes.

Replacement Level	WG Type	Specimen Properties	Observation on Compressive Strength Compared with Control Specimens	Ref.
100% precursor (<45 μm)	Mixed-color glass	Activator: 15 g of WG powder in 100 mL of 10 M NaOH Precursor: WG powder (<45 µm)	• 88 MPa compressive strength at 28 days	[115]
10–30% coal fly ash precursor (<20 μm)	Bottle glass	Activator: 4 M NaOH Precursors: 50% coal fly ash + 50% blast furnace slag Liquid/solid: 0.42	• 35% improved compressive strength at 7 days for 30% replacement level	[64]
10–20% slag precursor (specific surface area = 2009 cm^2^/g)	Liquid-crystal display glass	Activator: 5 M NaOH + water glass Precursor: slag Liquid/solid: 0.6	• The highest compressive strength was 53.46 MPa for 20% WG-based composite • 1.01%–1.07% higher compressive strength observed at 28 days	[67]

## Data Availability

Not applicable.
